# Structure of *Anabaena flos-aquae* gas vesicles revealed by cryo-ET

**DOI:** 10.1016/j.str.2023.03.011

**Published:** 2023-04-10

**Authors:** Przemys1aw Dutka, Lauren Ann Metskas, Robert C. Hurt, Hossein Salahshoor, Ting-Yu Wang, Dina Malounda, George J. Lu, Tsui-Fen Chou, Mikhail G. Shapiro, Grant J. Jensen

**Affiliations:** 1 Division of Chemistry and Chemical Engineering, California Institute of Technology, Pasadena, CA 91125, USA; 2 Division of Biology and Biological Engineering, California Institute of Technology, Pasadena, CA 91125, USA; 3 Division of Engineering and Applied Science, California Institute of Technology, Pasadena, CA 91125, USA; 4 Proteome Exploration Laboratory, Beckman Institute, California Institute of Technology, Pasadena, CA 91125, USA; 5 Howard Hughes Medical Institute, Pasadena, CA 91125, USA; 6 College of Physical and Mathematical Sciences, Brigham Young University, Provo, UT 84602, USA; 7 Present address: Biological Sciences Department, Purdue University, West Lafayette, IN 47907, USA; 8 Present address: Chemistry Department, Purdue University, West Lafayette, IN 47907, USA; 9 Present address: Department of Bioengineering, Rice University, Houston, TX 77005, USA; 10 Lead contact

## Abstract

Dutka et al. used cryo-ET supported by biochemical data and computational modeling to reveal the conserved structure of *Anabaena flos-aquae* gas vesicles. The resulting model gives insights into the distinctive mechanical properties of gas vesicles and their assembly.

## SUMMARY

Gas vesicles (GVs) are gas-filled protein nanostructures employed by several species of bacteria and archaea as flotation devices to enable access to optimal light and nutrients. The unique physical properties of GVs have led to their use as genetically encodable contrast agents for ultrasound and MRI. Currently, however, the structure and assembly mechanism of GVs remain unknown. Here we employ cryoelectron tomography to reveal how the GV shell is formed by a helical filament of highly conserved GvpA subunits. This filament changes polarity at the center of the GV cylinder, a site that may act as an elongation center. Subtomogram averaging reveals a corrugated pattern of the shell arising from polymerization of GvpA into a β sheet. The accessory protein GvpC forms a helical cage around the GvpA shell, providing structural reinforcement. Together, our results help explain the remarkable mechanical properties of GVs and their ability to adopt different diameters and shapes.

## Graphical abstract

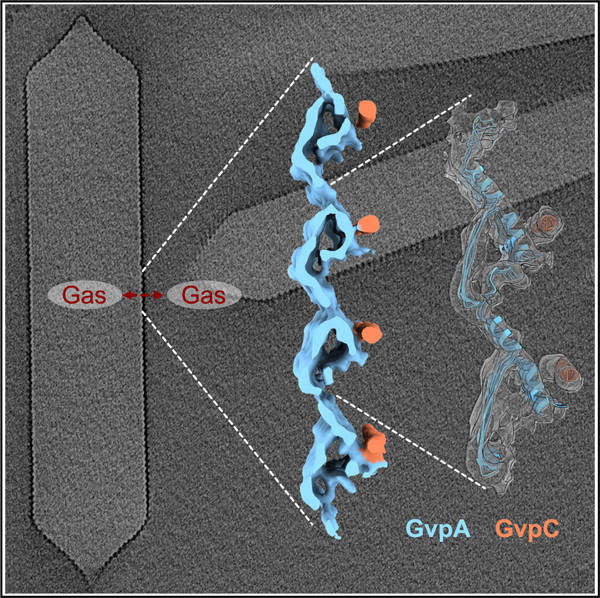

## INTRODUCTION

**Figure 1.**
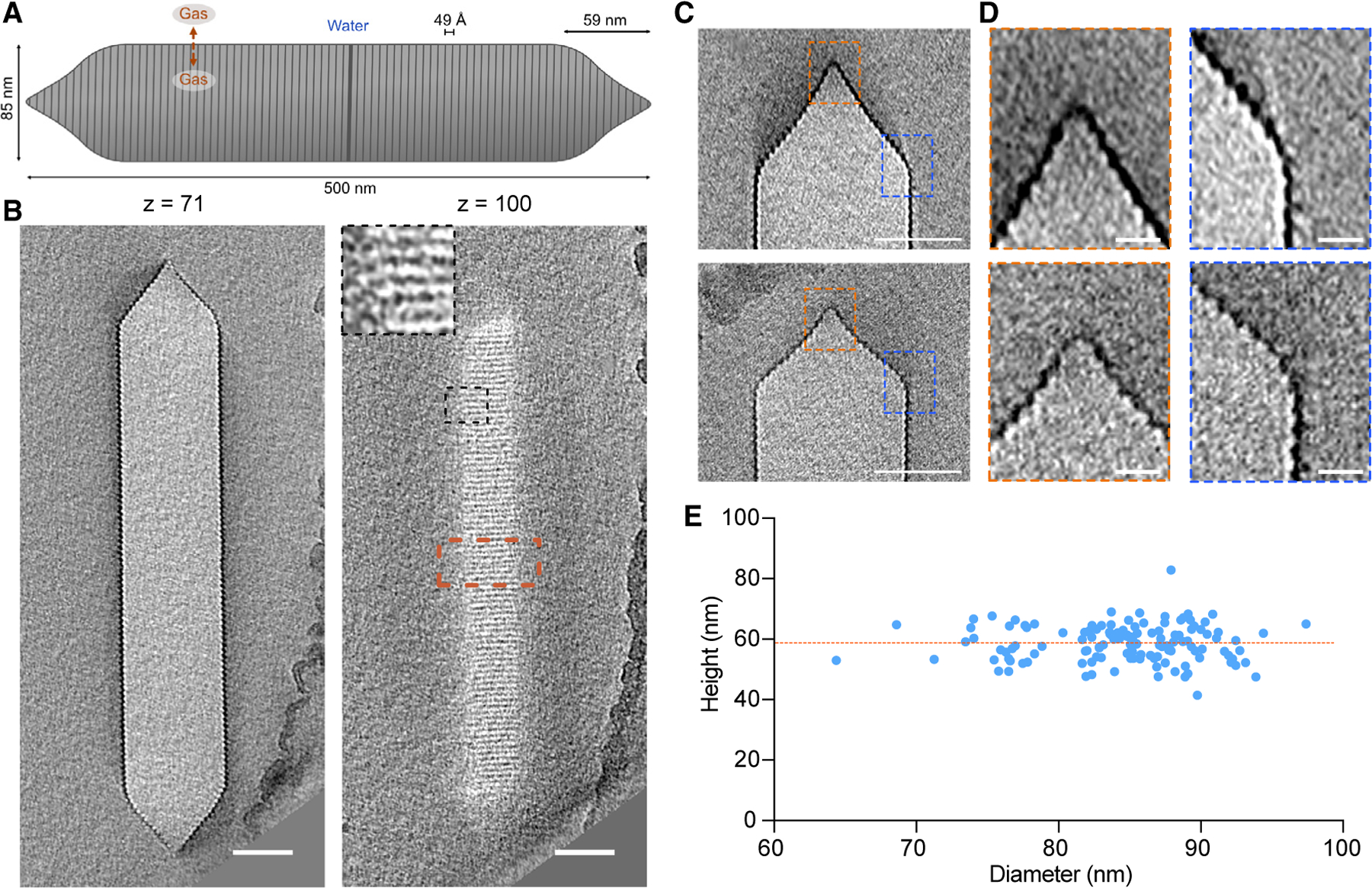
Molecular architecture of Ana GVs (A) Schematic of an Ana GV with dimensions annotated. (B) Representative slices at the indicated z heights from cryo-ET of an individual GV. Inset: enlargement of the area indicated by the black dashed box. Scale bars, 50 nm. (C) Central tomography slices of two conical GV ends with different morphologies. Scale bars, 50 nm. (D) Enlarged views of the areas indicated by orange (apex) and blue (cone to cylinder transition) dashed boxes in (C). Scale bars, 10 nm. (E) Distribution of the diameters and heights of conical GV ends; n = 132. The orange dashed line indicates the average height of the cones (59 ± 6 nm).

A fundamental property of many living organisms is their ability to move within their environment, with single-celled organisms capable of swimming, swarming, and aligning with magnetic fields. The molecular machines underlying many of these motility functions have been characterized in detail.^1–3^ However, the structure underlying one of the oldest evolved forms of motility, flotation, remains more mysterious. Some cyanobacteria, heterotrophic bacteria, and archaea regulate their buoyancy in aquatic environments to access sunlight and nutrients using intracellular flotation devices called gas vesicles (GVs).^4,5^ These unique protein nanostructures consist of a gas-filled compartment, typically ~100 nm in diameter and ~500 nm in length, enclosed by a ~3-nm-thick protein shell (Figure 1A) that can withstand hundreds of kilopascals of applied pressure.^6,7^ The interior of the shell is strongly hydrophobic, keeping out water while allowing gas molecules to diffuse in and out on a sub-millisecond timescale.^4,5^

In addition to their biological significance, GVs are a subject of intense interest for biotechnology. Analogous to fluorescent proteins, opsins, and CRISPR nucleases, GVs’ unusual biophysical properties can be harnessed for other purposes. The gaseous composition of GVs allows them to scatter ultrasound waves, enabling their use as genetically encoded reporters and actuators of cellular function deep in tissues.^8–14^ Other applications take advantage of GVs’ refractive index, gas permeability, and susceptibility to magnetic fields.^15–17^

GVs were discovered in the 19th century, but we still have limited knowledge of their structure and assembly. GVs adopt a cylindrical shape with conical caps (Figure 1A). Their components are encoded in operons containing relatively few genes (8–23+, depending on the species).^5^ One of these genes encodes the main structural protein, GvpA, a small (~8-kDa), highly hydrophobic protein that polymerizes to form the GV shell.^4^ In some species, the gene cluster contains a secondary structural protein called GvpC, which binds to the exterior of the shell to provide mechanical reinforcement.^18^ The remaining genes encode proteins whose functions are not well understood, possibly including chaperones, assembly factors, and additional minor shell constituents. GVs are nucleated as bicones that then elongate into a cylindrical shape with low-pitch helical ribs,^5,19^ but their detailed molecular structure is not known.

Here, we apply state-of-the-art cryoelectron tomography (cryo-ET) and subtomogram averaging techniques to GVs from the cyanobacterium *Anabaena flos-aquae* (Ana). These GVs are among the best studied by biophysicists^4,20,21^ and the most commonly used in biotechnology applications.^13,22,23^ We show that the Ana GV shell is formed by a continuous helical filament of repeating GvpA subunits, giving rise to a corrugated cylindrical structure with terminal cones that taper over a conserved distance. Near the middle of the cylinder, the angle of corrugation is inverted, suggesting a potential elongation center for GV biosynthesis. The corrugated shell is externally reinforced by circumferential rods of GvpC. Combining our cryo-ET data with an atomic model of the homologous *Bacillus megaterium* (Mega) GvpA protein determined in a complementary study,^24^ we build an integrative model of the Ana GV. This model explains the connection between the GV shell and GvpC and highlights the structural conservation of GVs between diverse species. Finally, we extend our study with biochemistry and computational modeling to corroborate our model and explore its implications for GV engineering.

## RESULTS

### Molecular architecture of GVs

Ana GVs are long, cone-tipped cylinders with diameters of 85 ± 4 nm^7^ and lengths of 519 ± 160 nm^6^ (Figures 1A and 1B). Although GVs have apparent helical symmetry, they are prone to deformation in thin ice (Figure S1) and are therefore intractable for cryoelectron microscopy (cryo-EM) helical processing. For this reason, we decided to use cryo-ET. However, cryo-ET analysis of GVs presents its own challenges. We observed that GVs are highly sensitive to electron dose, losing high-resolution features quickly before deflating and shrinking (Video S1). To mitigate this effect, we limited the total electron dose to ~45 electrons/Å^2^ per tilt series, which is ~2.5 times lower than typically used for high-resolution subtomogram averaging.^25,26^

We started by examining large-scale structural features. While the diameter and length of GVs have been characterized,^7,27^ the conical ends and their connection to the cylindrical body are less studied. Close inspection of individual caps in our cryo-tomograms revealed a heterogeneous morphology that deviated from a simple conical structure (Figures 1C and 1D). We observed two elements in the majority of cones: a pointed closed tip and a rounded transition region between the cone and cylinder (Figure 1D). The height of the conical caps was 59 ± 6 nm, independent of cylinder diameter (Figure 1E). The rounding of the base was more pronounced in GVs with larger diameters, so we also examined cryo-tomograms of Mega GVs, whose average diameter is ~30 nm smaller than that of Ana GVs. However, Mega GVs showed similar rounding at the cap transition (Figure S2), suggesting that this is a conserved feature of the structure independent of width.

### The GvpA spiral reverses polarity in the middle of the cylinder

**Figure 2.**
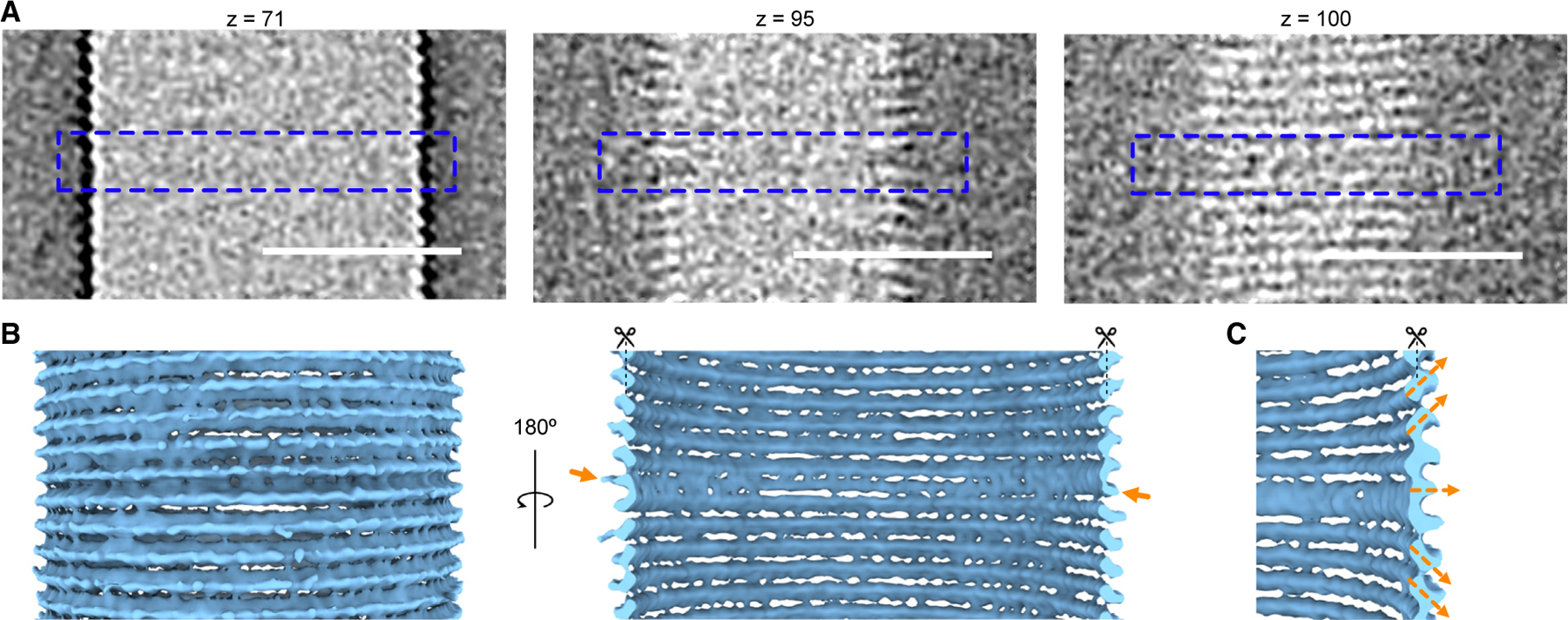
Polarity inversion point (A) Enlargement of the tomographic slices from Figure 1B (indicated by the orange dashed box) at different z heights. The blue dashed outlines indicate sections where polarity changes. Scale bars, 50 nm. (B) Subtomogram average of the middle region of the GV where the ribs reverse polarity. Arrows denote the rib where polarity is reversed. (C) Enlarged view of the subtomogram average in (B), highlighting the inversion of the helical assembly.

The GV shell consists of a low-pitch helix, running the length of the GV (Figures 2A and 2B). Near the middle of the GV, however, the angle of the helix abruptly inverts. Previously, Waaland and Branton^28^ noticed that one rib in the middle of the GV cylinder appears to be thicker than the others and suggested that this could be the growth point, where new GvpA subunits are added. Indeed, this abnormal rib was clearly visible in our tomograms (Figure 2A). To obtain a better understanding of the rib architecture in that region, we applied subtomogram averaging, which revealed that the angle of corrugation is opposite above and below the central rib (Figure 2B). This polarity inversion occurs within one rib, and the continuity of the spiral is not broken (Figures 2B and 2C). We were unable to distinguish whether the polarity of GvpA subunits changed relatively gradually within the space of one helical turn or abruptly from one monomer to the next. We also could not tell whether additional proteins are present at the inversion point.

By inspecting hundreds of cryo-electron micrographs of GVs from different species (Ana, Mega, and *Halobacterium salinarum*), we found that the polarity inversion point is a conserved feature (Figure S3). Although in general the inversion point was near the middle of the cylinder, in some cases it was located closer to one end (Figure S3A). If it is the nucleation point, then this suggests that GvpA subunits are not always added symmetrically in both directions. Additionally, we observed some examples where a GV exhibited different diameters on either side of the inversion point (Figure S3B). While we saw examples in all three species, it was most frequent and most pronounced in GVs from *H*. *salinarum* (Halo).

### Subtomogram averaging of the GV shell

**Figure 3.**
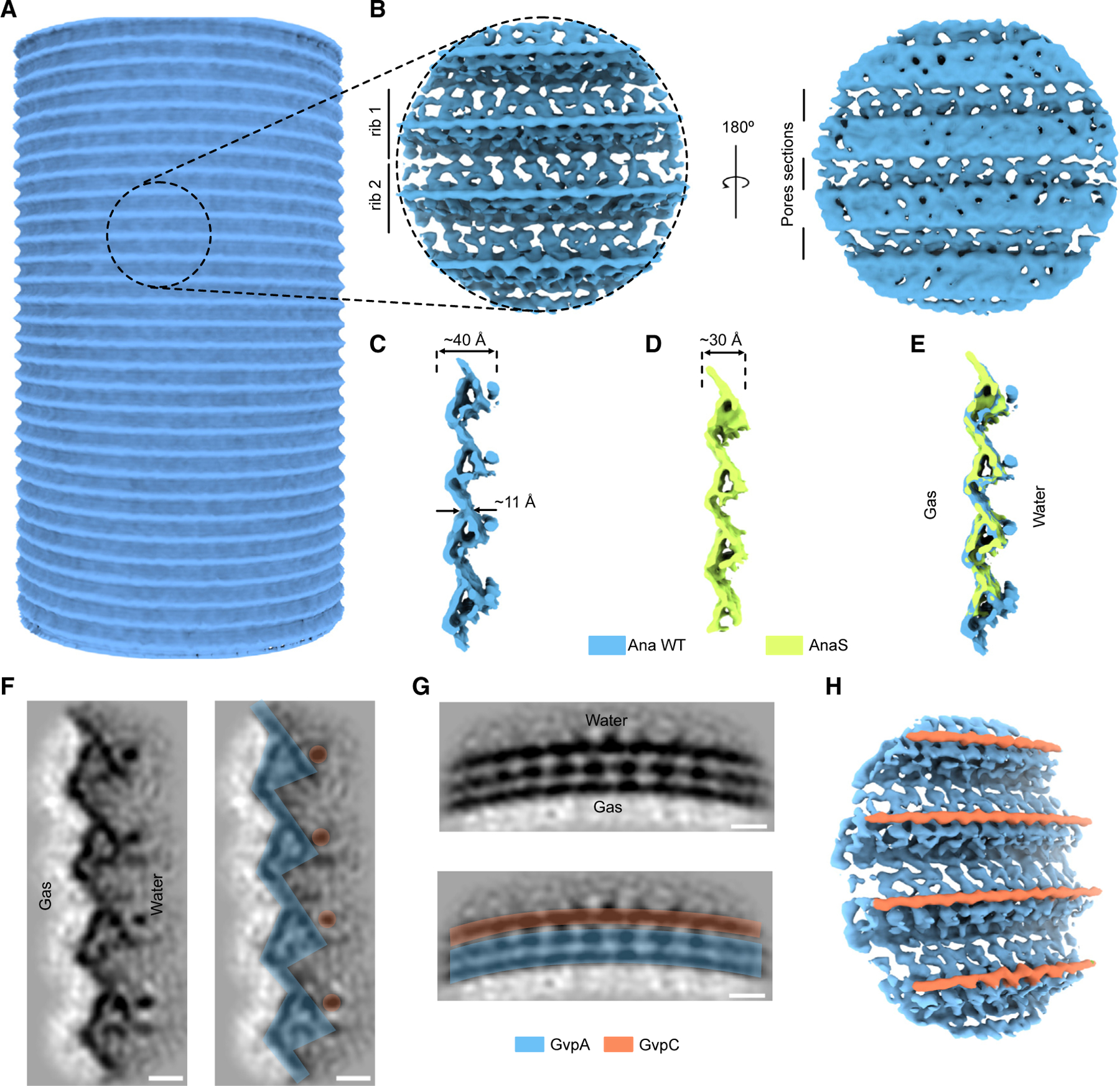
Cryo-ET structure of the Ana GV shell (A) Initial, low-resolution subtomogram average of a cylindrical GV segment. (B) Orthogonal views of a higher-resolution (7.7 Å) subtomogram average of the native Ana GV shell. (C–E) Cross-sections of the subtomogram averages of the GV shell: (C) native Ana GV, (D) AnaS GV, and (E) superimposed. (F and G) Projections trough the subtomogram average of the native Ana GV. (F) Projection along the GV helical axis. In the right panel color-coded densities corresponding to GvpA and GvpC. (G) Projection trough the neighboring subunits forming GV helix. Bottom: color-coded densities corresponding to GvpA and GvpC. Scale bars, 2 nm. (H) Segmented density map of the native Ana GV, indicating the locations of GvpC.

To understand the molecular details of the GV structure, we applied subtomogram averaging to the Ana GV shell in its native state and after biochemically removing the reinforcing protein GvpC to produce “stripped” (AnaS) GVs. Initially, we tried averaging tubular sections of the GVs. However, because of flattening and the low number of particles, the resolution of this approach was limited (Figure 3A). As an alternative, we decided to average only small sections of the shell with randomly seeded particle centers similar to an oversampling method.^25,29^ This strategy produced a higher number of particles and allowed more rigorous 3D classification to remove distorted particles. With this method, we produced subtomogram averages of native Ana (Figure 3B and S4) and AnaS (Figure S5) GV shells with global resolutions of 7.7 Å and 7.3 Å, respectively (Table S1; Figures S4 and S5). Despite high global resolution, our maps manifested a certain degree of anisotropy with significantly lower resolution in the y direction (Figures S4D and S5D). The particle poses after subtomogram averaging indicate that all particles are oriented outward and consistent with a helical arrangement (Figure S6). Typically, we observed one significant break in the particle poses per GV, which corresponds to the inversion point. However, because of the strong effects of missing wedge artifacts on tubular structures, such as GVs, they typically appear as two disconnected arches. As a result, we observed a fraction of misaligned particles in the direction of the missing wedge. Furthermore, flattening of the GV cylinder and small variability in diameters could lead to inaccurate alignment of some particles, resulting in blurring of the structure, particularly in the y direction, and limiting resolvability of the secondary structures. Although the GV corrugated structure has strong features in the x and z directions, there are no features in the y direction that could aid subtomogram alignment. A visual examination of the maps revealed that, despite the lower resolution, the map for the native Ana GV shell had higher quality (Figures 3F, 3G, and S4C). For this reason, we used the native GV shell map for further interpretation, and the AnaS map was only used to determine the position of GvpC.

The subtomogram average revealed a prominent pattern of beveled ribs, giving rise to the corrugated GV shell. The shell was ~4 nm wide at its thickest and only ~1 nm thick in the region between adjacent ribs (Figure 3C). We also observed pores in this region, at the interface between neighboring ribs of the spirals (Figure 3B), likely allowing gas to diffuse in and out of the GV. In contrast to the complex exterior face of the GV shell, the gas-facing interior appeared relatively smooth.

Comparing the maps of native Ana and AnaS GVs (lacking GvpC), we noticed a pronounced rod-like structure positioned along the GV ribs that is absent in AnaS (Figures 3C–3E). Previously, various models for GvpC binding to the GV shell have been proposed,^30^ with most of the field favoring one in which GvpC spans longitudinally across GvpA ribs.^13,31^ Our structure shows instead that GvpC binds circumferentially to the thickest part of the GV shell, creating a spiral cage around the GV cylinder (Figures 3F–3H). We do not yet know whether the GvpC filament binds the central inversion rib or extends to the conical caps, where the decreasing radius of curvature might be prohibitive, or whether it is continuous, as the average would blur away gaps.

### Conserved assembly of GvpA and its consequences on GV development and mechanics

**Figure 4.**
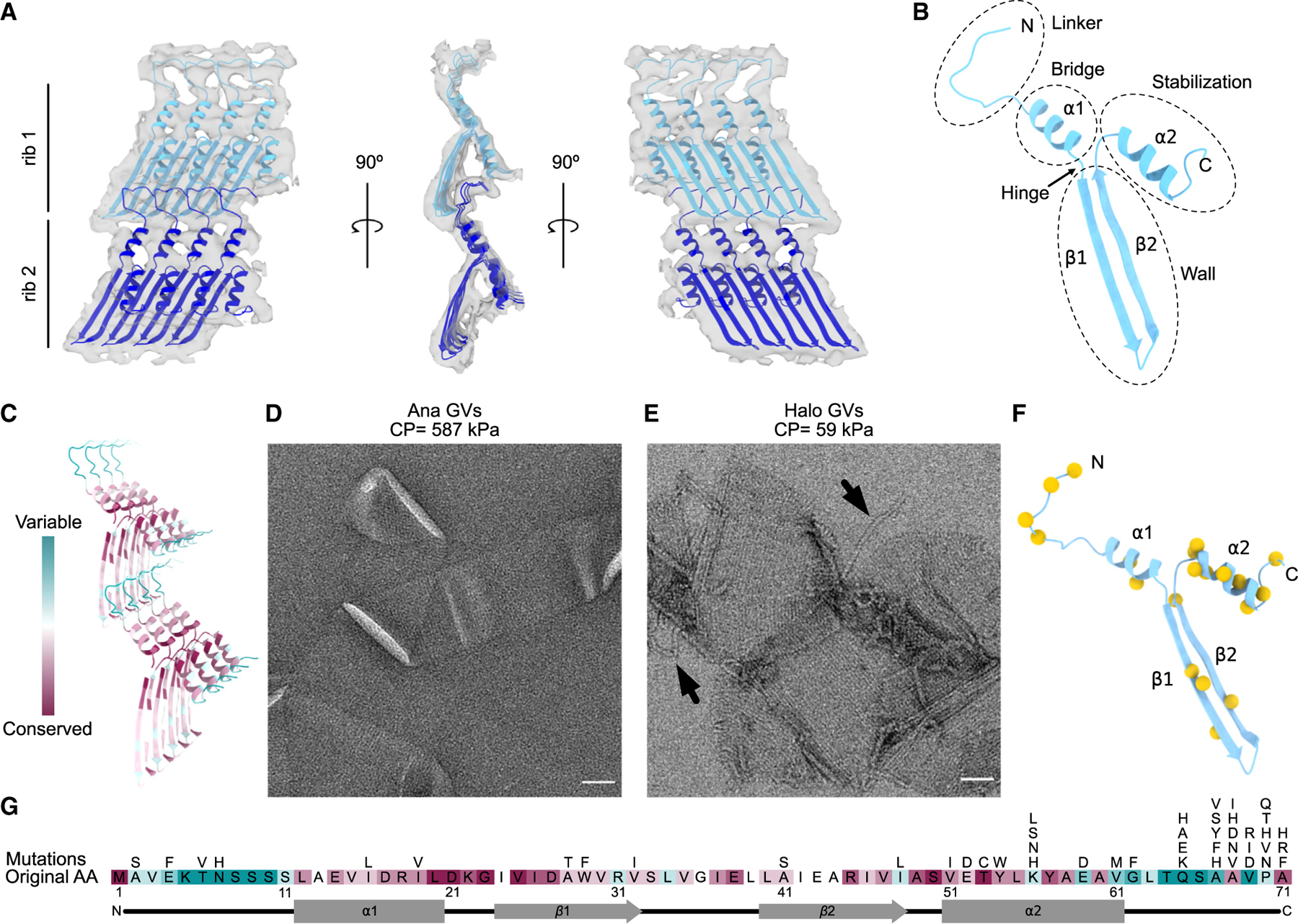
Conserved assembly of the GV shell (A) Segmented ~8-Å resolution structure of two adjacent GvpA ribs determined by subtomogram averaging (gray surface), fitted with a homology model of GvpA based on GvpA2 (PDB: 7R1C).^24^ (B) Domain annotation within an individual GvpA. (C) Conservation analysis of GvpA determined by ConSurf.^32^ (D and E) Negative-stain EM images of collapsed GVs from (D) Ana and (E) Halo. Arrows indicate separated GvpA filaments. Collapse pressure (CP) is indicated above. Scale bars, 50 nm. (F) Location of tolerated mutation sites (yellow spheres) in the GvpA structure (blue). (G) Map of all tolerated mutations in GvpA. Original sequence colored by conservation score as in (C).

The resolution of our Ana GV density map was sufficient for rigid-body fitting of a homology model of GvpA. Taking advantage of the high degree of conservation of the protein, we used the structure of GvpA2 from Mega solved by helical reconstruction in a contemporaneous study.^24^ The only substantial difference between GvpA from Ana and Mega is an extended C terminus in the latter (Figure S7), so our homology model was complete and fit well into our cryo-ET density map (Figures 4A and S8). After docking the model to our map, we observed that the fit of α helices is not perfect. It could be due to the limited resolution of our maps or because these helices adopt a slightly different conformation compared with Mega GvpA2. The GvpA spiral is formed by polymerization of individual subunits, resembling the packing of amyloids. All domains of the small GvpA protein play a role in building the GV shell (Figure 4B), packing into a tight structure with only small pores contributing to the remarkable stability of GVs; we find that purified GVs are stable for years at cool or ambient temperature.

As mentioned above, the only major difference between Mega GvpA2 and Ana GvpA is the presence of an elongated C terminus (Figure S7). This C terminus was not resolved in a recent structure solved by helical processing,^24^ presumably because of its flexibility. In our cryo-ET of Mega GVs, we observed additional density on the surface of the shell that is absent from the structures of AnaS and native Ana GV shells (Figure S9). The density was not highly regular but appeared connected. It may be that this extra density belongs to the C terminus of GvpA2, which perhaps plays a role in stabilizing the GV shell.

The sequence of GvpA, the major structural protein, is highly conserved in all GV-producing species,^33,34^ and we think it is likely that its structure is similarly conserved, as evidenced by our ability to fit a model from Mega GvpA2^24^ into the density of Ana GvpA. Remarkably, though, GvpA can assemble into GVs with varying diameters (Figure S10A)^7^ and morphologies (Figures S10B and S10C). For instance, the largest Halo GVs are ~7 times larger in diameter than the smallest Mega GVs. One key to understanding different morphologies may lie in what appears to be a hinge region located between helix α1 and strand β1 (Figure 4B), where a conserved glycine resides (Figures 4G and S7). Small sequence differences in GvpA have been suggested to contribute to different morphologies of GVs.^4^ Halo contains two independent GV gene clusters, p-vac and c-vac.^5^ The sequences of the GvpA encoded by the two clusters are 94% identical (Figure S7), but these cluster can produce GVs with a lemon shape (Figure S10B) or a more typical cylindrical shape with conical caps (Figure S10C).

We used ConSurf^32^ to visualize the evolutionary conservation of GvpA, revealing that the most conserved residues are located in the β sheets and α helices (Figure 4C). In contrast, the N-terminal domain of the protein responsible for interactions between neighboring ribs showed the greatest variability (Figure 4C). Within the generally conserved β strands, the most variable sites were those interacting with the N terminus from the subunit below. This variability in amino acid composition in the domains responsible for holding adjacent ribs together might be one factor contributing to differences in the mechanical strength of GVs. Under hydrostatic pressure, GVs can collapse, forming flattened sacs.^7^ The critical pressure required to collapse GVs varies greatly between species. For example, the hydrostatic collapse pressure threshold of Ana GVs is 587 kPa, while that of Halo GVs is 59 kPa, an order of magnitude lower.^6^ By EM imaging, we found that Ana GVs collapse without major disruptions to the rib structure (Figure 4D), while collapsed Halo GVs often exhibit major disruption of the rib structure and separation of the GvpA filament (Figure 4E). This supports the idea that the strength of connectivity between ribs varies between species.

To test the importance of conserved GvpA residues in GV assembly, we mapped tolerated mutations by screening a scanning site saturation library of GvpA mutants in *Escherichia coli* engineered to express a hybrid gene cluster encoding the structural proteins GvpA and GvpC from the Ana GV gene cluster and the accessory proteins from the Mega GV gene cluster. GV-producing mutant clones were identified by nonlinear X-wave ultrasound (xAM) (Figures 4F, 4G, and S11). The results largely correlated with observed evolutionary conservation, with the highest number of function-retaining mutations occurring in the evolutionarily variable C-terminal coil (Figure 4G). Interestingly, the only conserved region that tolerated mutations well was helix α2, which is not involved in interactions between monomers but plays a crucial role in GvpC binding (see below).

### GvpC forms a helical spiral around the GV shell

**Figure 5.**
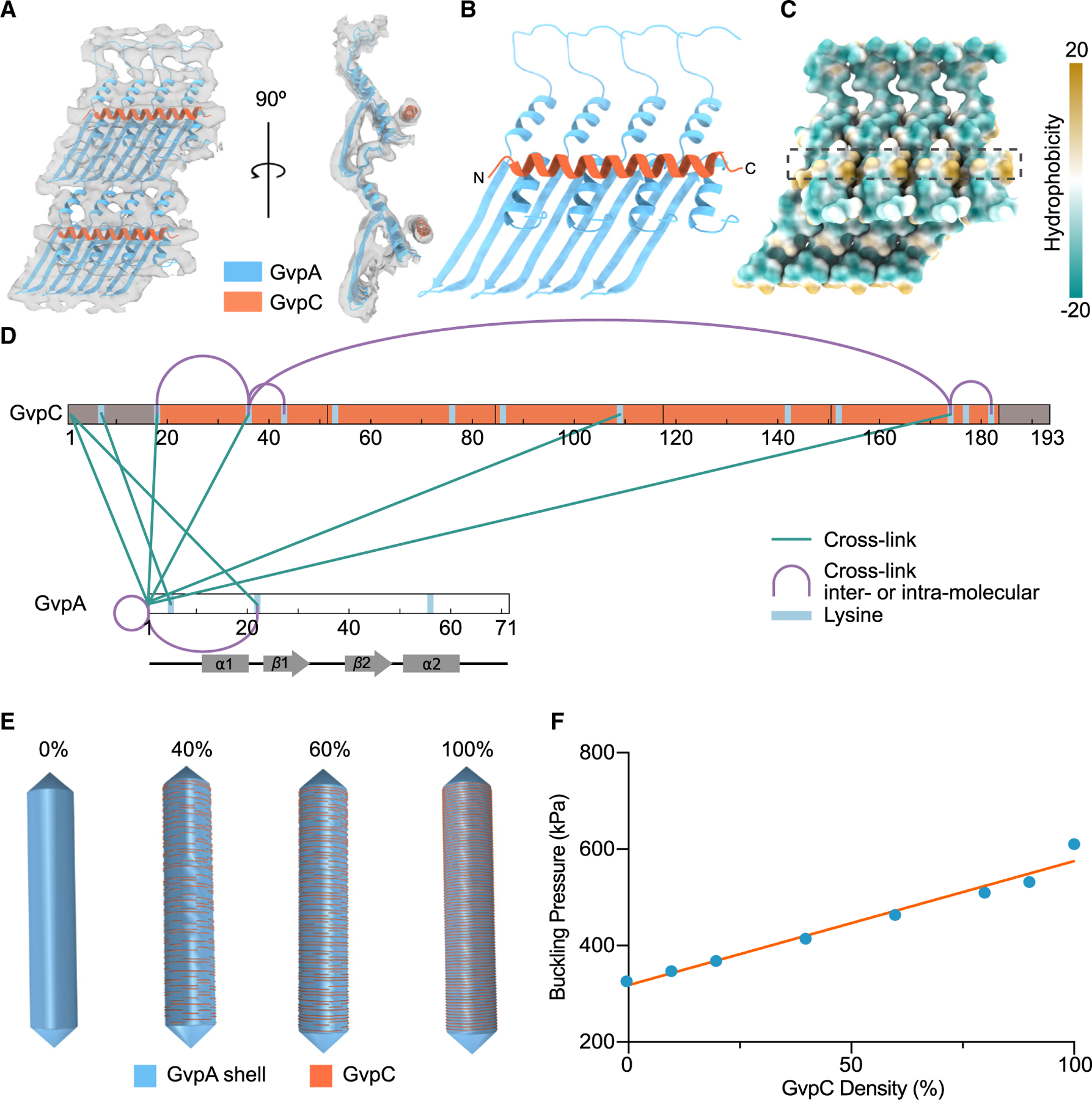
Mechanical reinforcement of the GV shell by GvpC (A) Segmented ~8-Å resolution subtomogram average of neighboring Ana GvpA monomers connected by GvpC (gray surface) fitted with a model of GvpA and a poly-Ala chain corresponding in length to one repeating sequence of GvpC. (B) Resulting GvpC binding model. (C) GvpC binding site (dashed black box) at the hydrophobic pockets between α2 helices of GvpA. The surface of GvpA is colored by hydrophobicity. (D) Cross-linked sites between GvpA and GvpC identified by mass spectrometry. (E) Finite element shell models of a GV with a length of 500 nm and width of 85 nm and the indicated degree of GvpC saturation. (F) Buckling pressure as a function of GvpC density. The orange line represents a simple linear regression fit.

Having identified GvpC in our subtomogram average of the Ana GV shell (Figure 3H), we next investigated how GvpC binds to GvpA and how multiple GvpC proteins might cooperate to strengthen GVs. GvpC is predicted to form an amphipathic α-helical structure composed of a characteristic 33-residue repeating sequence^4,35,36^ (Figure S12A). Ana GvpC consists of 5 such repeats plus short N and C termini. To build a model of a GV shell decorated with GvpC, we fitted a poly-alanine helix of a length corresponding to one repeating unit into our subtomogram average (Figures 5A and 5B).

We found that GvpC binds perpendicular to the surface-exposed α2 helices of GvpA, directly above the hydrophobic pockets (Figures 5B and 5C). Although there is insufficient density to anchor the helix, we predict that GvpC binds to GvpA with its hydrophobic side facing the shell. In addition to being amphipathic, GvpC also has an unequal distribution of charge (Figure S12B). In our model, GvpC binds directly above the negatively charged C terminus of GvpA (Figure S12C). One 33-residue repeating sequence of GvpC interacts with approximately four GvpAs, indicating a GvpC to GvpA ratio of at least 1:20 when saturated. This is close to the previously calculated ratio of 1:25.^30^

Despite multiple rounds of 3D classification and application of different focus masks, we were unable to resolve the junctions between neighboring GvpC molecules. Instead, GvpC appeared as a continuous helical belt. To get a better understanding of GvpC-GvpC and GvpC-GvpA interactions, we performed chemical cross-linking coupled with mass spectrometry (XLMS) (Table S2). Most of the cross-links we observed were between the N terminus of GvpA and apparently random locations on GvpC (Figure 5D), which is consistent with the close association between the N terminus of GvpA and the GvpA α2 helix in the adjacent rib, where GvpC binds, in our structure (Figure 5A). However, we did not observe any cross-links between GvpC and helix α2, potentially because of the unfavorable orientation of the lysines. Among GvpC-GvpC cross-links, the most interesting was between K36 and K174 (Figure 5D). The distance between these residues is ~20 nm, too far for an intramolecular cross-link,^37^ suggesting that GvpC termini are either closely packed or potentially interact head to tail (Figure S13).

To quantify the effect of increasing GvpC occupancy on GV stabilization, we used solid mechanics simulations to estimate the applied pressure at which the GV shell starts to buckle, a parameter relevant to its ability to withstand hydrostatic pressure as well as produce nonlinear signal in ultrasound imaging. We implemented several finite element models of a GV shell, each 500 nm in length and 85 nm in diameter and with a custom density of GvpC molecules. From a continuous belt, representing 100% GvpC, we randomly removed GvpC-length (25-nm) segments of the helix to achieve the desired saturation for each model (Figure 5E). We subjected the outer surface of each GV shell to uniform normal stress, simulating hydrostatic or acoustic pressure, and obtained a critical buckling pressure by linear buckling analysis. We observed a simple linear dependence of buckling on scaffolding protein density (Figure 5F), consistent with previous experimental findings that GvpC level can be utilized to modulate the GV buckling threshold.^22^

## DISCUSSION

The GV shell has remarkable mechanical properties; despite being only ~3 nm thick, it is highly stable and can withstand up to hundreds of kilopascals of pressure. This is achieved by tight packing of the GvpA subunits into a low-pitch helix that forms a corrugated cylinder. On the macroscopic level, corrugation is typically used when flexibility is important (e.g., pipes) or to increase durability and strength (e.g., unpressurized cans). One or both of these properties might be similarly important for GV function. Our data indicate that GV cylinders can be significantly deformed without collapsing the structure.^7^ This elasticity of the GV shell may be crucial for adapting to pressure fluctuations *in vivo* and enables GVs to be used as contrast agents in high-specificity nonlinear ultrasound imaging.^38^ We noticed a highly conserved glycine between helix α1 and strand β1 of GvpA. The single hydrogen in the side chain of glycine gives it much more flexibility than other amino acids,^39^ suggesting that this region may act as a hinge that confers elasticity on the shell structure and lets it adapt to different geometries, such as those observed in terminal cones or the bodies of lemon-shaped GVs.

The primary contact between adjacent GvpA subunits is mediated by lateral interactions of antiparallel β strands in an extended sheet, resembling the aggregation of β-amyloids.^40,41^ Such assemblies are typically stabilized by an extensive network of backbone hydrogen bonding, conferring outstanding strength.^42^ Such strength is also observed in GVs from diverse species; individual GvpA monomers can only be dissociated from the polymer by harsh chemical treatment.^43,44^ That backbone interactions are the main force driving subunit polymerization is consistent with the wide range of diameters observed in different species;^7^ as the curvature of the cylinder changes, the relative positions of backbone residues will be affected much less than those of side chains. We find that GvpA domains involved in forming the GV wall have a low tolerance for mutations, likely because of selective pressure to preserve the highly hydrophobic composition of the β sheets and maintain interactions with the linker domain connecting subsequent coils of the helix. Our scanning mutagenesis data largely correlate with results obtained for Halo GVs.^45^ Interestingly, however, Halo GVs appear to be more tolerant to mutations in the conserved regions, possibly because, unlike Ana or Mega GVs, they are synthesized without turgor pressure in the cells.

Stacked ribs of the continuous GvpA polymer are joined by interactions of the coiled N termini from one row of subunits with the β strands of the subunits in the next. We observe that the strength of these inter-rib interactions varies between species, likely related to evolutionary variability in the N-terminal linker. It has been observed previously that the critical collapse pressure of Mega GVs is much higher than that of Ana or Halo GVs,^6^ likely because of the narrower diameter of Mega GVs.^46–48^ However, we note that the C terminus of Mega GvpA is longer than in other species, and in our tomograms of Mega GVs, we observed extended irregular surface densities connecting ribs. We suggest that these extra densities correspond to the extended C termini of Mega GvpA2 and may confer additional mechanical strength.

Other mechanisms also enhance the strength of the GV shell. Almost all GV gene clusters encode an additional, minor structural protein, GvpC, that binds to the GvpA helical spiral and reinforces the shell;^22,49^ we find that GvpC binds to the surface-exposed α2 helix of GvpA. In our mutational analysis, this helix was relatively mutation tolerant, suggesting that it has a minimal role in GvpA shell integrity and instead acts primarily as an adapter for GvpC. In contrast to previous models of GvpC spanning ribs, we find that GvpC instead tracks along ribs, forming a spiral cage around the GV cylinder. Our XLMS results indicate close conjunction of GvpC molecules, and even with multiple masking and 3D classification strategies, we never observed discontinuity in the GvpC rod in our subtomogram averages. Although we could not resolve interactions between GvpC N and C termini, we showed previously that their removal leads to a significant drop in critical collapse pressure of Ana GVs.^22^ Here, we used finite element simulations to quantify the reinforcing effect of GvpC density on GV buckling and find that the degree of strengthening is directly proportional to the amount of GvpC bound. However, full GvpC occupancy is not required for full strengthening, and small gaps in the GvpC cage have a negligible effect on collapse pressure. Even though our work focused on Ana GVs, it is possible that the GvpC binding model is conserved between different species of GVs. Previously, the interaction between GvpA and GvpC was studied in Halo by split-GFP assay^50^ providing similar results to those obtained in our XLMS analysis.

In the initial stage of assembly, GVs grow as bicones until reaching their target diameter; at that point, growth elongates the central section, producing cylinders that can reach several micrometers in length.^5,10^ The trigger for this transition is unclear. Our data show that the height of mature cones is relatively constant, regardless of GV diameter, indicating that the number of helical turns/height is the measured quantity rather than the number of GvpA subunits. Our observation of a polarity inversion near the middle of the GV suggests that this is the site of cylinder elongation, with individual subunits being incorporated in both directions. In some cases, we observed that the elongation center was located closer to one end of the GV, suggesting a mechanism that does not require GvpA subunits to be added symmetrically in both directions. Although GV cylinders typically exhibit a uniform diameter, we documented some examples with different diameters on either side of the elongation center. We observed variations in the shape of conical ends within and between GVs. This suggests that mismatches in GV geometry might arise in the initial bicone growth stage, but further investigation is needed to fully dissect the mechanism of GV morphogenesis.

Currently, the method of choice for solving the structure of helical assemblies is helical reconstruction.^51,52^ However, the large and nonuniform diameter of Ana GVs (~85 nm) and their susceptibility to deformation during cryopreservation present challenges for this approach. Cryo-ET and subtomogram averaging can circumvent these limitations by focusing on smaller and therefore more uniform 3D sections of the object of interest. Subtomogram averaging can reach high resolution in certain favorable cases, such as for large^53^ or symmetrical^26,54^ proteins, but for most targets, resolution has remained limited. Here we show that even with a fairly challenging target, recent developments in cryo-ET data collection and subtomogram averaging methods combined with integrative modeling make it possible to obtain a sufficient resolution to dock an atomic model. Our work, together with a complementary study of Mega GVs,^24^ advances our understanding of the molecular architecture of GVs and may inform further engineering of GVs to serve as genetically encoded contrast agents and biosensors.

### Limitations of the study

Using subtomogram averaging, we determined the structure of the Ana GV protein shell, providing insight into GV morphogenesis and explaining their unusual mechanical properties. Because of the high conservation of GvpA, we were able to build an integrative model of the Ana GV shell using the homologous structure of Mega GvpA2.^24^ However, the limited resolution of our map only allowed rigid-body fitting. Despite the high homology of GvpA, there might be a subtle difference between the structure of GvpAs from different organisms, reflecting unique proprieties of each GV type. Additionally, we are not able to discern whether there are any conformational changes caused by GvpC binding. Future higher-resolution studies will be necessary to allow for flexible fitting of GvpA models to extend our knowledge on GV evolution and mechanics. Additionally, a better understanding of how GVs are assembled will require biochemical and structural work focusing on the GV initiation and elongation process.

## STAR★METHODS

### RESOURCE AVAILABILITY

#### Lead contact

Further information and requests for resources and reagents should be directed to and will be fulfilled by the lead contact, Grant J. Jensen (grant_jensen@byu.edu).

#### Materials availability

This study did not generate new unique reagents.

#### Data and code availability

The unprocessed tilt series used for the data analysis are available upon request. Representative tomograms for Ana, Mega, and Halo GVs have been deposited in the Electron Microscopy Data Bank under accession codes EMDB: EMD-29922, EMD-29925, EMD-29924, EMD-29923. Subtomogram averages for native Ana and AnaS GV shell have been deposited in EMDB under accession codes EMD-29921 and EMD-29916, respectively. The integrative model of Ana GvpA/GvpC has been deposited in the Protein Data Bank (PDB): 8GBS. The XLMS data have been deposited to the ProteomeXchange Consortium with the dataset identifier PXD038631. The code for ultrasound data collection and processing is available upon request.

### EXPERIMENTAL MODEL AND SUBJECT DETAILS

GVs were produced either in native sources, *Anabaena flos-aquae* (Ana) and *Halobacterium salinarum* NRC1 (Halo), or expressed heterologously in Rosetta 2(DE3)pLysS *Escherichia coli*, *Bacillus megaterium* (Mega). We followed previously published protocols by Lakshmanan et al.^6^ describing in details bacterial growth conditions specific for production of each GV type investigated here.

### METHOD DETAILS

#### GV preparation

GVs were isolated as previously described.^6^ In the final steps of buoyancy purification, the sample buffer was exchanged for 10 mM HEPES, pH 7.5. To obtain GVs stripped of GvpC (AnaS), 6 M urea solution was added to purified native GVs and two additional rounds of buoyancy purification were performed. AnaS GVs were subsequently dialyzed in 10 mM HEPES, pH 7.5. Concentrations were measured by optical density (OD) at 500 nm using a spectrophotometer (NanoDrop ND-1000, Thermo Scientific).

#### Cryo-ET

A freshly purified GV sample was diluted to OD_500_ = ~20 (Ana and Halo), ~3 (AnaS), or ~1 (Mega) and mixed with 10 nm BSA-coated gold beads. A 3 μL volume of sample was applied to C-Flat 2/2 – 3C grids (Protochips) that were freshly glow-discharged (Pelco EasiGlow, 10 mA, 1 min). GV samples were frozen using a Mark IV Vitrobot (FEI, now Thermo Fisher Scientific) (4°C, 100% humidity, blot force 3, blot time 4 s).

Tilt-series were collected on a 300 kV Titan Krios microscope (Thermo Fisher Scientific) equipped with a K3 6k × 4k direct electron detector (Gatan). Multi-frame images were collected using SerialEM 3.39 software^55^ using a dose-symmetric tilt scheme. Super-resolution movies were acquired at a pixel size of 0.8435 Å (53,000× magnification) with varying defocus from - 1.0 to - 3.5 μm. Tilt-series of Halo and Mega GVs were collected from −60° to 60° with 3° increments. Tilt-series of native Ana GVs were collected in two sessions. The first set was collected from −60° to 60° with 3° increments and the second from −44° to 44° with 4° increments. For AnaS GVs, data were collected from −45° to 45° with 3° increments. Due to the rapid shrinking of GVs during exposure to the electron beam (Video S1), the total accumulated dose in all cases was limited to 45 electrons/Å^2^. Data collection parameters are summarized in Table S1.

Raw movies were binned by a factor of 2 and gain- and motion-corrected on-the-fly using Warp.^56^ Assembled tilt-series were exported to Dynamo^57^ for automated alignment using *autoalign_dynamo*.^65^ Aligned tilt-series were CTF corrected and full tomograms were either reconstructed in Warp with a pixel size of 10 Å or manually aligned and reconstructed using Etomo.^66^

#### Subtomogram averaging - inversion point

Sub-volume extraction, alignment, and averaging were performed using the Dynamo software package.^57^ Particles for subtomogram averaging of the inversion site were manually selected from GVs with a diameter of ~85 nm, yielding a total of 68 particles. Sub-volumes were extracted from 4x binned tomograms with a final pixel size of 6.748 Å and 180x180x180 box size. The initial reference for particle alignment was generated by averaging segments with azimuth-randomized orientations. Due to the low number of particles, subtomogram averaging was not performed according to a gold standard. Instead, convergence of the structure was analyzed by changes in particle shifts and cross-correlation scores. During the final rounds of refinement, a soft cylindrical mask was applied to the central 40% of the GV tube.

#### Subtomogram averaging - GV shell

Subtomogram averaging was carried out using Dynamo,^57^ Warp,^56^ Relion-3.1,^58^ and M,^53^ software packages. Data transfer between Dynamo and Warp/M was carried out using a set of tools provided by *warp2catalogue* and *dynamo2m*.^65^ Particle selection and initial reference generation were performed using the Dynamo package. Orientations and positions of shell sections were determined using geometrical tools for particle picking in Dynamo.^67^ Initial estimates of positions and orientations on the GV shell were generated with an interparticle distance of ~150 Å (~3 ribs). Particles were extracted in Dynamo with a pixel size of 10 Å and averaged. After removal of duplicated particles, data was transferred to Warp and subtomograms were reconstructed with a pixel size of 5 Å based on the alignment information from Dynamo. Subtomograms were subsequently refined in RELION, re-reconstructed at 2.5 Å /pixel and 3D classified without alignment in RELION. After 3D classification, several additional rounds of 3D refinement were carried out in RELION. Finally, subtomograms were reconstructed at 1.687 Å /pixel and iteratively refined in RELION and M using a soft-edged mask around ~3 or 4 adjacent ribs. Although we did not see a resolution boost after iterative refinement of the tilt-series parameters in M, subsequent refinement in RELION produced a better-quality reconstruction when applied to particles reconstructed after M refinement. Final maps were post-processed in RELION. The resolution was estimated using a soft-edged mask around ~3–4 adjacent ribs in 3DFSC program.^64^ The final results are summarized in Figures S4, S5, and Table S1.

#### Model building and validation

Although the density map for AnaS reached a higher overall resolution, individual features were better resolved in the map of native Ana GVs (Figure S4), so all model building was performed using this map. To build the GvpA model, a high-resolution cryo-EM structure of the homologous GvpA2 from *B. megaterium* (PDB: 7R1C)^24^ was fitted into the segmented cryo-ET density map corresponding to an individual subunit in UCSF Chimera.^60^ The GvpA amino acid sequence was rebuilt by manual replacement of mismatched residues in Coot.^63^ The *A. flos-aquae* GvpA model was subsequently refined by rigid-body fitting using the Phenix real-space refinement tool.^62^ The refined GvpA model was used to populate a larger section of the cryo-ET map in UCSF Chimera.^60^ The multimeric GvpA model was further refined by rigid-body fitting in Phenix to maximize fit into the density map. The GvpC model was built as a poly-Ala chain in Coot. The poly-Ala chain corresponds in length to a single 33-residue repeating sequence of GvpC and spans across four subunits of GvpA.

The quality of the fit was analyzed by visual inspection and fitting scores from UCSF Chimera (Figure S8). We roughly placed four GvpA subunits at the height of one rib and performed a global search using “fitmap” command in Chimera. Subsequently, we analyzed scores for cross-correlation and fraction inside density for each fit. The three best results with similar fitting scores all fit our density map very well and are only different in that they shift by one subunit along Y (the are essentially all the same “fit”). We obtained similar results with a starting point at the height of other ribs.

#### Negative stain EM

To prepare collapsed GV samples, the purified GV sample was diluted to OD_500_~ 0.5 and pressurized in a sealed syringe until the solution turned transparent. Three microliters of the target sample was applied to a freshly glow-discharged (Pelco EasiGlow,15 mA, 1 min) Formvar/carbon-coated, 200 mesh copper grid (Ted Pella) for 1 min before blotting. Afterward, the sample was incubated for 1 min with a 0.75% uranyl for-mate solution before blotting and air-dried. Image acquisition was performed using a Tecnai T12 (FEI, Thermo Fisher Scientific) EM at 120 kV, equipped with a Gatan Ultrascan 2 k×2 k CCD.

#### Cross-linking mass spectrometry (XLMS)

The cross-linking procedure was carried out according to the manufacturer’s instructions (Thermo Fisher). In brief, a freshly purified sample of native Ana GVs in 10 mM HEPES, pH 7.5 was mixed with an excess of cross-linker: either DSSO or BS3 (Thermo Fisher). The sample was incubated for 1h at room temperature and subsequently the reaction was quenched with Tris buffer at a final concentration of 20 mM.

The crosslinking samples were digested in an S-Trap mcrio spin column (Protifi, USA) according to the manufacturer’s instructions. For trypsin digestion, an additional aliquot of trypsin was added after 24 hours on the S-trap column and the digestion continued for another 24 hours. After elution and drying, peptides were suspended in LCMS-grade water containing 0.2% formic acid and 2% acetonitrile for further LC-MS/MS analysis. LC-MS/MS analysis was performed with an EASY-nLC 1200 (Thermo Fisher) coupled to a Q Exactive HF hybrid quadrupole-Orbitrap mass spectrometer (Thermo Fisher). Peptides were separated on an Aurora UHPLC Column (25 cm × 75 μm, 1.6 μm C18, AUR2-25075C18A, Ion Opticks) with a flow rate of 0.35 μL/min for a total duration of 43 min and ionized at 1.7 kV in the positive ion mode. The gradient was composed of 6% solvent B (2 min), 6–25% B (20.5 min), 25–40% B (7.5 min), and 40–98% B (13 min); solvent A: 2% ACN and 0.2% formic acid in water; solvent B: 80% ACN and 0.2% formic acid. MS1 scans were acquired at a resolution of 60,000 from 375 to 1500 *m*/*z*, AGC target 3e6, and a maximum injection time of 15 ms. The 12 most abundant ions in MS2 scans were acquired at a resolution of 30,000, AGC target 1e5, maximum injection time 60 ms, and normalized collision energy of 28. Dynamic exclusion was set to 30 s and ions with charges +1, +7, +8, and >+8 were excluded. The temperature of the ion transfer tube was 275°C and the S-lens RF level was set to 60. For cross-link identification, MS2 fragmentation spectra were searched and analyzed using Sequest and XlinkX node bundled into Proteome Discoverer (version 2.5, Thermo Scientific) against *in silico* tryptic digested *Dolichospermum-flos-aquae* GvpA from the Uniprot database. The maximum missed cleavages were set to 2. The maximum parental mass error was set to 10 ppm, and the MS2 mass tolerance was set to 0.05 Da. Variable crosslink modifications were set DSS (K and protein N-terminus, +138.068 Da) for BS3 crosslink and DSSO (K and protein N-terminus, +158.004 Da) for DSSO crosslink, respectively. For BS3 crosslink, the dynamic modifications were set to DSS hydrolyzed on lysine (K, +156.079 Da), oxidation on methionine (M, +15.995 Da), protein N-terminal Met-loss (−131.040 Da), and protein N-terminal acetylation (+42.011 Da). For the DSSO crosslink, the dynamic modifications were set to DSSO hydrolyzed on lysine (K, +176.014 Da), DSSO Tris on lysine (K, +279.078 Da), oxidation on methionine (M, +15.995 Da), protein N-terminal Met-loss (−131.040 Da) and protein N-terminal acetylation (+42.011 Da). Carbamidomethylation on cysteine (C, +57.021 Da) was set as a fixed modification. The false discovery rate (FDR) for crosslinked peptide validation was set to 0.01 using the XlinkX/PD Validator Node and crosslinks with an Xlinkx score greater than 30 were reported here. The raw data have been deposited to the ProteomeXchange Consortium^68^ via the PRIDE^69^ partner repository.

#### Scanning site saturation library generation and screening

The scanning site saturation library was constructed via a Gibson assembly-based version of cassette mutagenesis as previously described.^70^ Briefly, the *A*. *flos-aquae* GvpA coding sequence was divided into sections that tiled the gene, and oligos were designed to have a variable middle region with flanking constant regions against which PCR primers with Gibson overhangs were designed. The variable region was designed to sequentially saturate each residue with every amino acid other than the WT at that position, plus a stop codon to produce truncation mutants (*i*.*e*., the size of such libraries is 20 * [# of amino acids in the protein]). Oligos were synthesized as a pool by Twist Biosciences, and were amplified by 10 cycles of PCR (both to make them double-stranded and to add overhangs for Gibson assembly) using Q5 polymerase (according to the manufacturer’s protocol, but with 5 μM of each primer) and assembled with the rest of the GV gene cluster (*i*.*e*., Ana GvpC and Mega GvpR-GvpU) into a pET28a vector via Gibson assembly using reagents from New England Biolabs. Assembled libraries were electroporated into NEB Stable *E*. *coli* and grown in Lennox LB with 100 mg/μL kanamycin and 1% glucose.^71^ Plasmid DNA was miniprepped (Econospin 96-well filter plate, Epoch Life Science) and verified by Sanger sequencing. Ultrasound-based phenotyping of mutants was performed in BL21-AI (Thermo Fisher) as previously described,^23^ and all screened mutants were sequenced using the evSeq pipeline.^72^

#### Finite element simulation

We first developed a finite element model of a single stripped GV isolated from *A*. *flos-aquae* (AnaS). The geometry, adapted from the cryo-EM images, comprises a cylindrical shell with conical ends, with height and diameter, respectively, of 500 nm and 85 nm. The protein wall was idealized as a continuum shell with a thickness of 2.8 nm and a shell density of 1350 kg/m^3^. The rib-like structure of the gas vesicle wall was mirrored in the computational model by an elastic anisotropic material model, with elastic moduli across and along the principal axis of the GV of 0.98 GPa and 3.92 GPa, respectively.^38^ In order to simulate the nearly incompressible nature of the protein shell, we assigned a Poisson’s ratio of 0.499. We note that the material parameters were not obtained from direct experimental measurements, but rather chosen such that, in addition to falling within a range of parameters consistent with those of protein-based biological materials,^73^ they effectively replicated the buckling pressures observed experimentally.

We next added a helical rod that spirals around the cylindrical portion of the GV shell, modeling the GvpC molecules. We modeled the GvpC rod as a shell of radius 0.6 nm. The helical structure was generated by assigning a pitch of 4.9 nm. The finite element model of the resultant wild-type GV was obtained by discretizing the entire geometry with quadrilateral shell elements of effective side length 1 nm with reduced integration (*i*.*e*., S4R elements) in Abaqus (Dassault Systemes Simulia, France). These general-purpose shell elements with only one integration point within each element are capable of capturing both tensile and in-plane bending, and, with a sufficiently fine mesh size, are computationally cost-effective. We subjected the interior surfaces of the GV to an initial pressure of 101 kPa, modeling the inner gas pressure. We further subjected the vertices at both the top and bottom conical ends of the GV to a zero-displacement Dirichlet boundary condition, which prevented rigid body translations and rotations of the entire GV structure.

In order to investigate the effect of GvpC density on the buckling pressure, we first computed the total length of the helix where N, D, and z are the total number of turns, the perimeter of the GV cross-section, and the pitch of the helix, respectively. Given the pitch and the length of the cylindrical segment of the GV model, 416.5 nm, the total number of turns was computed as 85. We thus computed the total length of the helix as 22.702 micrometers. Given that the length of GvpC is ~25 nm, about 908 GvpC molecules constituted the helix in our model. We generated six additional finite element models with distinct GvpC saturation levels of 90%, 80%, 60%, 40%, 20%, and 10%, for which we randomly removed about 90, 180, 360, 540, 720, and 810 GvpC units, respectively.

We conducted linear buckling analysis (LBA) and solved the corresponding eigenvalue problem to obtain the threshold buckling pressures for each model. We solved this problem using the Lanczos algorithm and obtained the first ten modes of buckling. Unlike the buckling modes (*i*.*e*., eigenvectors), which were virtually identical at different levels of GvpC saturation, the buckling pressures (*i*.*e*., eigenvalues) were remarkably dependent on the GvpC density, with an almost linear monotonic relation, where decreasing the saturation level decreases the buckling pressure. Figure S14 depicts the buckling modes and pressures for 100%, 60%, 20%, and 0% GvpC saturations.

#### Bioinformatics and visualization

Protein sequence alignment was carried out using Clustal Omega^74^ and visualized with Jalview.^75^ Protein conservation analysis was performed using ConSurf.^32^ Data were visualized using GraphPad Prism, IMOD,^59^ Chimera,^60^ and ChimeraX.^61^ Identified crosslinks were visualized using xiNET.^76^

### QUANTIFICATION AND STATISTICAL ANALYSIS

The heights of the GVs’ conical ends (Figure 1E) were manually measured from cryo-electron tomograms using ImageJ. The average height is calculated from 132 conical ends and reported as mean ± SD.

The subtomogram averages were determined using software listed in the key resources table. Details of the data processing are displayed in Figures S4 and S5, and Table S1. The resolution anisotropy and final FSC curves (Figures S4D and S5D) were determined using the 3DFSC package.

The ultrasound data (Figures 4F and S11), XLMS analysis (Figure 5D and Table S2), and finite element simulation (Figures 5E, 5F, and S14) were analyzed using software listed the key resources table.

No other statistical analyses were performed.

## Supplementary Material

MMC2

MMC1

## Figures and Tables

**Table:** KEY RESOURCES TABLE

REAGENT or RESOURCE	SOURCE	IDENTIFIER
Bacterial and virus strains		

*E. coli* Rosetta 2(DE3)pLysS	Millipore Sigma	Cat# 71401–3
*E. coli* BL21-AI	Thermo Fisher Scientific	Cat# C607003
*E. coli* NEB Stable	New England Biolabs	Cat# C3040H
*Dolichospermum flos-aquae* strain 1403/13F	SAMS LIMITED CCAP	Cat# 1403/13F
Halobacterium sp. NRC-1, Living, Plate	Carolina Biological Supply	Cat# 154801

Chemicals, peptides, and recombinant proteins		

Q5 High-Fidelity DNA Polymerase	New England Biolabs	Cat# M0491L
NEBuilder HiFi DNA Assembly Master Mix	New England Biolabs	Cat# E2621L
LB Broth (Lennox)	Sigma-Aldrich	Cat# L3022–1KG
Kanamycin sulfate	Sigma-Aldrich	Cat# K1377–25G
D-(+)-Glucose	Sigma-Aldrich	Cat# G7021–5KG
SoluLyse^™^ Bacterial Protein Extraction Reagent	Genlantis	Cat# L200500
Cyanobacteria BG-11 Freshwater Solution	Sigma-Aldrich	Cat# C3061
20x PBS, Sterile	Teknova	Cat# P1225
Lysozyme from chicken egg white	Sigma-Aldrich	Cat# L6876
IPTG, Isopropyl β D 1 thiogalactopyranoside	Teknova	Cat# I3305
Chloramphenicol	Sigma-Aldrich	Cat# C0378
Ampicillin sodium salt	Sigma-Aldrich	Cat# A0166
Tris-HCl 1 M, pH 7.5	Teknova	Cat# T1075
Sodium chloride (NaCl)	Sigma-Aldrich	Cat# S7653
Magnesium sulfate heptahydrate	Sigma-Aldrich	Cat# 230391
Casein hydrolysate	Sigma-Aldrich	Cat# 22090
Gibco^™^ Bacto^™^ Yeast Extract	Gibco	Cat# 212750
Sodium nitrate	Amresco	Cat# 0598
Potassium phosphate monobasic	Sigma-Aldrich	Cat# P5655
Sodium metasilicate nonahydrate	Sigma-Aldrich	Cat# S4392
Sodium carbonate	Sigma-Aldrich	Cat# 71345
Sodium bicarbonate	Sigma-Aldrich	Cat# S5761
Citric acid	BDH Chemicals	Cat# BDH7397
EDTA, disodium salt dihydrate	Alfa Aesar	Cat# A15161
Ferric ammonium citrate	Amresco	Cat# 0846
Urea	Sigma-Aldrich	Cat# 51456
Trisodium citrate dihydrate	Sigma-Aldrich	Cat# S1804
Potassium chloride	Sigma-Aldrich	Cat# 746436
D-Sorbitol	Sigma-Aldrich	Cat# S6021
Formic Acid, LC/MS Grade	Fisher Scientific	Cat# A11750
Acetonitrile, LC/MS Grade	Fisher Scientific	Cat# A9554
Water, LC/MS Grade	Fisher Scientific	Cat# W64
Trypsin, TPCK Treated	ThermoFisher Scientific	Cat# 20233
DSSO, crosslinker	ThermoFisher Scientific	Cat# A33545
BS3, crosslinker	ThermoFisher Scientific	Cat# A39266

Deposited data		

Cryo-electron tomogram for Ana GV (Figures 1 and 2A)	This study	EMD-29922
Cryo-electron tomogram Mega GV (Figure S2A)	This study	EMD-29925
Cryo-electron tomogram Halo GV, p-vac (Figure S9B)	This study	EMD-29924
Cryo-electron tomogram Halo GV, c-vac (Figure S9C)	This study	EMD-29923
Subtomogram average of the native Ana GV shell	This study	EMD-29921
Subtomogram average of AnaS GV shell	This study	EMD-29916
Integrative model of Ana GvpA/GvpC	This study	PDB 8GBS
XLMS data	This study	PXD038631
Atomic model of GvpA2	Huber et al.^24^	PDB 7R1C

Oligonucleotides		

Primers for Gibson assembly	Integrated DNA Technologies	N/A
Mutagenic oligo pool	Twist Bioscience	N/A

Recombinant DNA		

pST39 plasmid containing pNL29 Mega GV gene cluster	Addgene	Cat# 91696

Software and algorithms		

SnapGene	SnapGene	https://www.snapgene.com/
MATLAB	Mathworks	https://matlab.mathworks.com/
Abaqus	Dassault Systmes	https://www.3ds.com/products-services/simulia/products/abaqus/
SerialEM	Mastronarde^55^	https://bio3d.colorado.edu/SerialEM/
Warp	Tegunov and Cramer^56^	http://www.warpem.com/warp/
Dynamo	Castaño-Díez et al.^57^	https://wiki.dynamo.biozentrum.unibas.ch/w/index.php/Main_Page
RELION	Zivanov et al.^58^	https://relion.readthedocs.io/en/release-3.1/
M	Tegunov et al.^53^	http://www.warpem.com/warp/
IMOD	Kremer et al.^59^	https://bio3d.colorado.edu/imod/
UCSF Chimera	Pettersen et al.^60^	https://www.cgl.ucsf.edu/chimera/index.html
UCSF ChimeraX	Goddard et al.^61^	https://www.rbvi.ucsf.edu/chimerax/
Phenix	Adams et al.^62^	https://phenix-online.org/
Coot	Emsley et al.^63^	https://www2.mrc-lmb.cam.ac.uk/personal/pemsley/coot/
3DFSC	Tan et al.^64^	https://github.com/nysbc/Anisotropy
dynamo2m	Burt el al.^65^	https://github.com/alisterburt/dynamo2m
autoalign_dynamo	Burt el al.^65^	https://github.com/alisterburt/autoalign_dynamo
Xcalibur	ThermoFisher Scientific	https://www.thermofisher.com/us/en/home.html
Proteome Discoverer	ThermoFisher Scientific	https://www.thermofisher.com/us/en/home.html
XlinkX node for Proteome Discoverer	ThermoFisher Scientific	https://www.thermofisher.com/us/en/home.html

Other		

C-flat Holey Carbon R2/2 300 Mesh	EMS	Cat# CF-223C-100
Formvar/Carbon 200 mesh, Copper	Ted Pella	Cat# 01801
Vitrobot Mark IV	FEI, Thermo Fisher Scientific	https://www.thermofisher.com/us/en/home/electron-microscopy/products/sample-preparation-equipment-em/vitrobot-system.html
Gatan K3 summit camera with post-column energy filter	Gatan	https://www.gatan.com/products/tem-imaging-spectroscopy/k3-cameras
300 kV Titan Krios microscope	FEI, Thermo Fisher Scientific	https://www.thermofisher.com/us/en/home/electron-microscopy/products/transmissionelectron-microscopes.html
Tecnai T12 120 kV	FEI, Thermo Fisher Scientific	Out-of-production
EconoSpin 96-Well Filter Plate	Epoch Life Sciences	Cat# 2020–001
Shaker incubator with cooling, CO2 control and illumination	Infors HT	Multitron II
Benchtop microcentrifuge	Eppendorf	Cat# 5430R
UltraCruz Filter Flask, PES, 0.22	Santa Cruz Biotechnology	Cat# sc-359029
1000 mL Filter System, PES Filter Material, 0.22 μm	CELLTREAT	Cat# 229708
EMD Millipore^™^ Steritop Threaded Bottle Top Filter	EMD Millipore	Cat# S2GPT05RE
STERICUP-GP500	EMD Millipore	Cat# S2GPU10RE
NanoDrop 2000C series	Thermo Scientifc	Cat# ND-2000C
1-cm-path-length quartz cuvette	Hellma Analytics	Cat# 176.700-QS
18G BLUNT NEEDLE 1.5IN STERILE	VWR International	Cat# 89134–020
96 well filter plate	Bio Basic	SD5006
Square Petri Dish with Grid	VWR	60872–310
S-Trap micro spin column	PROTIFI	Cat# C02-micro-80
Aurora UHPLC Column, 25 cm × 75 μm, 1.6 μm C18	Ion Opticks	Cat# AUR2–25075C18A-CSI
Q Exactive HF hybrid quadrupole-Orbitrap mass spectrometer	ThermoFisher Scientific	N/A
Easy-nLC 1200	ThermoFisher Scientific	LC140

## Highlights

- Gas vesicles (GVs) are formed by ~3-nm corrugated protein shells
- Corrugation reverses at the cylinder midpoint, which may act as an elongation center
- The protein shell is primarily formed by the conserved major structural protein GvpA
- GvpC provides extra reinforcement by forming a helical spiral around the GV cylinder
