# Supplementary material for: Structure of Anabaena flos-aquae gas vesicles revealed by cryo-ET
Source: Structure. Author manuscript; Available in PMC 2023 May 15. (PMC10185304; doi:10.1016/j.str.2023.03.011)
Supplement: MMC1 [file NIHMS1887368-supplement-MMC1.pdf]

Structure, Volume *31*

Supplemental Information

Structure of *Anabaena flos-aquae* gas vesicles revealed by cryo-ET

Przemysław Dutka, Lauren Ann Metskas, Robert C. Hurt, Hossein Salahshoor, Ting-Yu Wang, Dina Malounda, George J. Lu, Tsui-Fen Chou, Mikhail G. Shapiro, and Grant J. Jensen

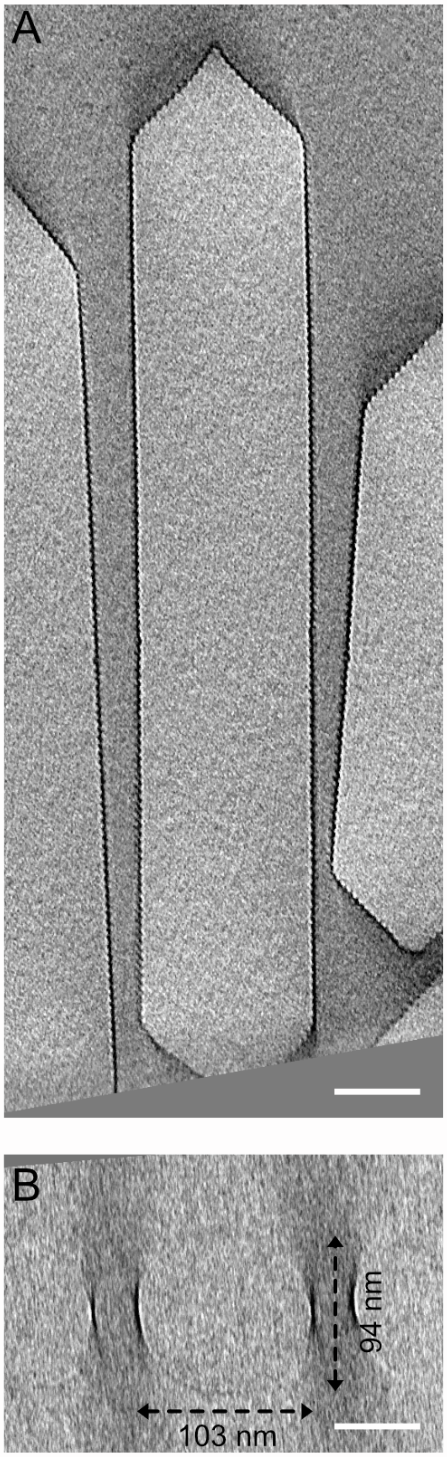

Figure S1. GV flattening in the thin ice, related to Figure 1 and STAR Methods. (**A**) XY and (**B**) XZ tomographic slices of the deformed Ana GV. Scale bars, 50 nm.

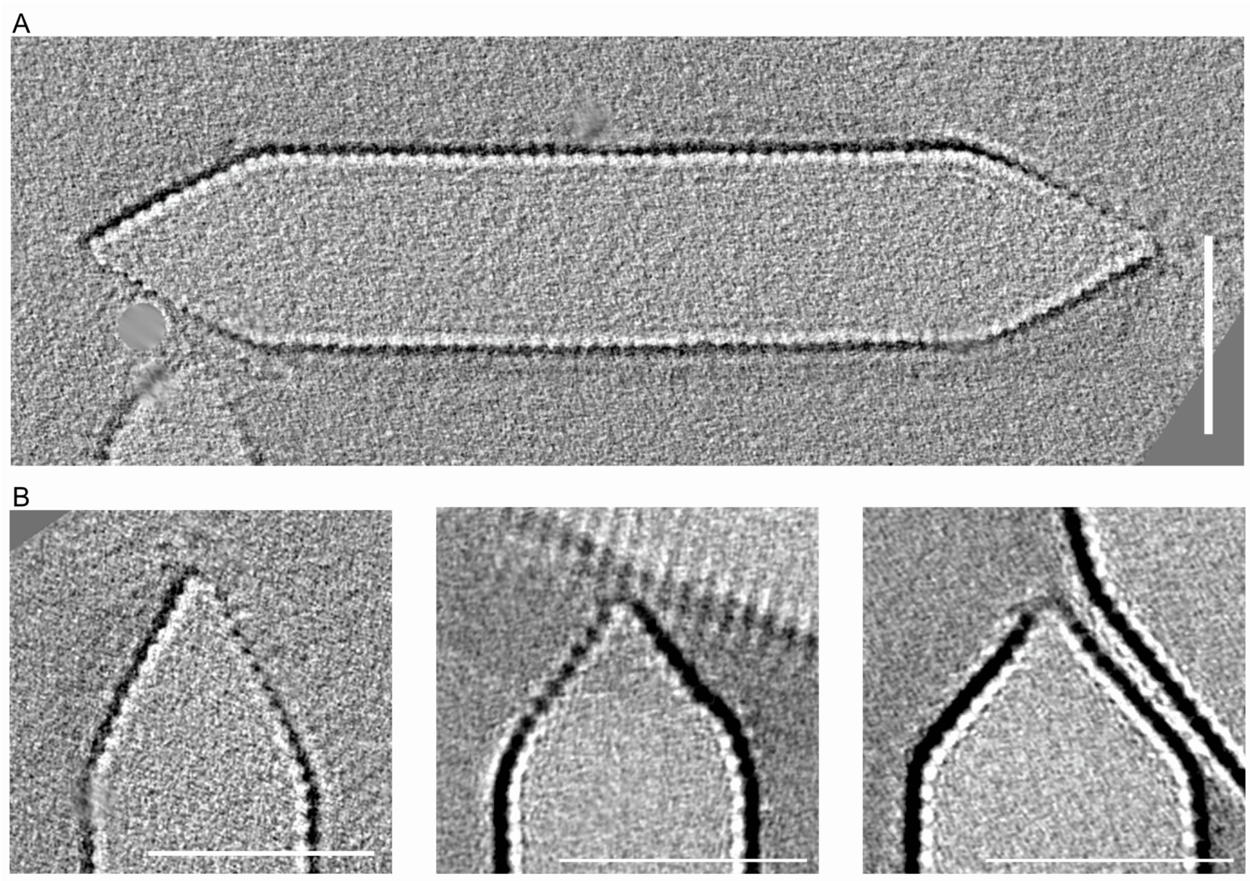

Figure S2. The architecture of Mega GVs, related to Figure 1. (**A**) Representative central slice from cryo-electron tomogram of individual Mega GV. (**B**) Central tomographic slices of the Mega GV conical ends with slightly different shapes. Scale bars, 50 nm.

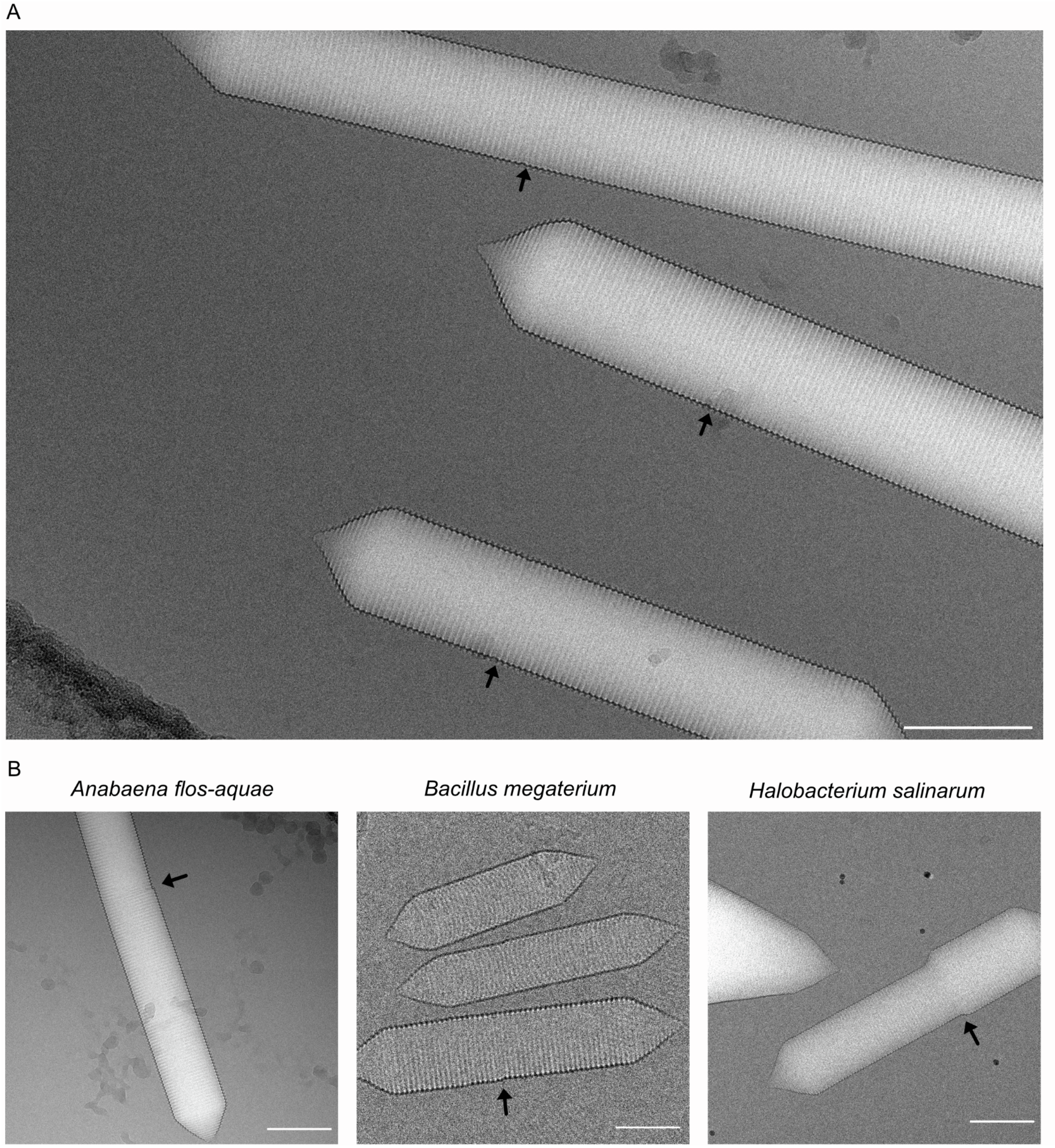

Figure S3. GV Polarity inversion point, related to Figure 2. (**A**) Location of the polarity inversion point. (**B**) GVs can have different diameters on either side of the inversion point. The black arrows indicate the location of the inversion point.

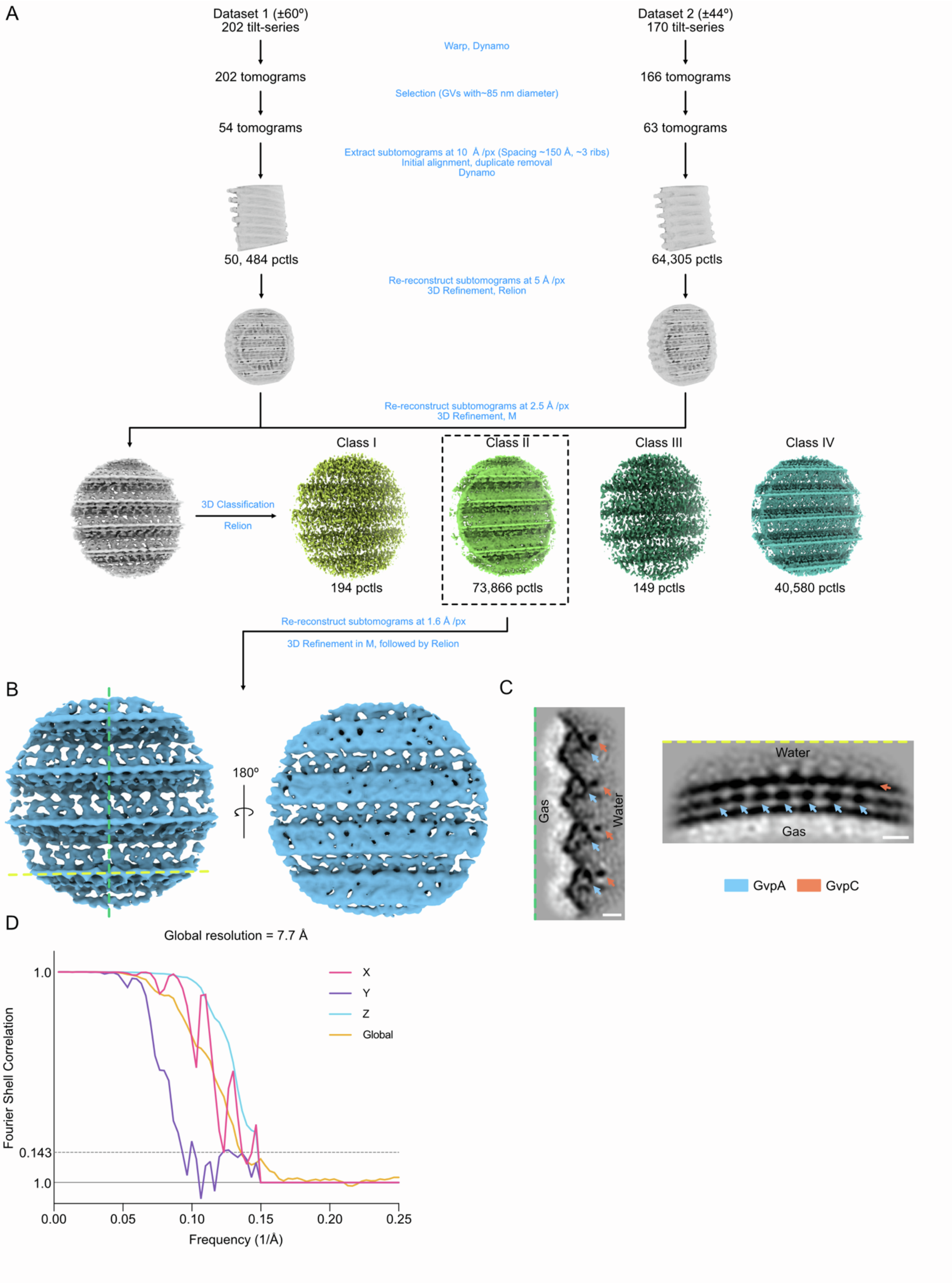
A
Dataset 1 (±60°)
202 tilt-series
Dataset 2 (±44°)
170 tilt-series
Warp, Dynamo
202 tomograms
166 tomograms
Selection (GVs with~85 nm diameter)
54 tomograms
63 tomograms
Extract subtomograms at 10 Å /px (Spacing ~150 Å, ~3 ribs)
Initial alignment, duplicate removal
Dynamo
50, 484 pctls
64,305 pctls
Re-reconstruct subtomograms at 5 Å /px
3D Refinement, Relion
Re-reconstruct subtomograms at 2.5 Å /px
3D Refinement, M
3D Classification
Relion
Class I
194 pctls
Class II
73,866 pctls
Class III
149 pctls
Class IV
40,580 pctls
Re-reconstruct subtomograms at 1.6 Å /px
3D Refinement in M, followed by Relion
B
180°
C
Gas
Water
Water
Gas
GvpA
GvpC
D
Global resolution = 7.7 Å
Fourier Shell Correlation
1.0
0.143
1.0
0.00
0.05
0.10
0.15
0.20
0.25
Frequency (1/Å)
X
Y
Z
Global

Figure S4. CryoET data processing for the native Ana GV shell, related to Figure 3 and STAR Methods. (**A**) Simplified schematic of the subtomogram averaging workflow highlighting crucial steps in the pipeline. (**B**) Orthogonal views of the final average. (**C**) Gray-scale orthographic slices of the native Ana GV shell in the positions indicated by the green and yellow dashed lines in **B**. Orange and blue arrows point to GvpC rod and GvpA subunits, respectively. (**D**) Global FSC curve for AnaS GV shell (yellow), and map anisotropy analysis by FSC curves in X (pink), Y (purple) and Z (blue) directions.

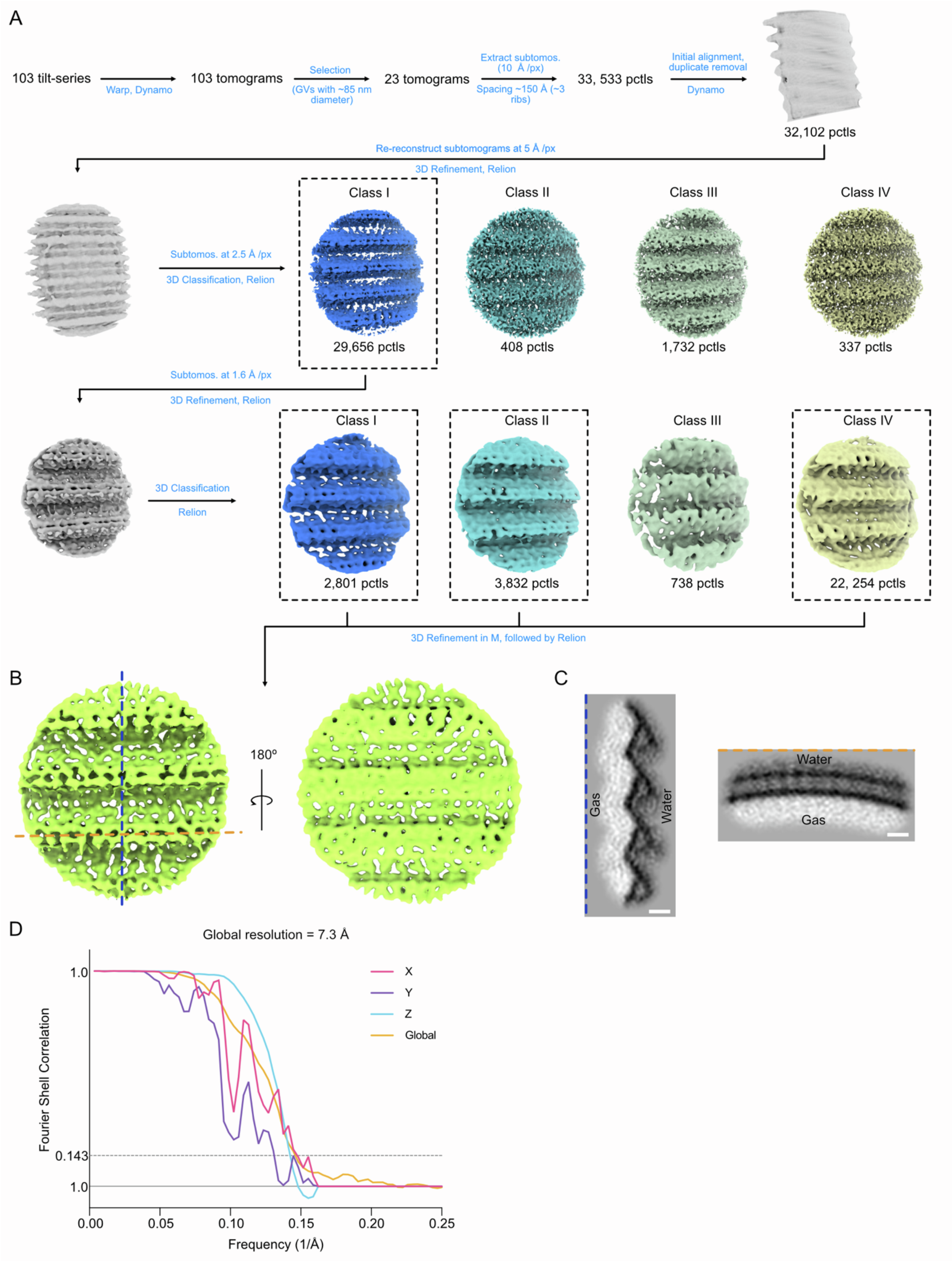
A
103 tilt-series
Warp, Dynamo
103 tomograms
Selection
(GVs with ~85 nm diameter)
23 tomograms
Extract subtomos. (10 Å /px)
Spacing ~150 Å (~3 ribs)
33, 533 pctls
Initial alignment, duplicate removal
Dynamo
32,102 pctls
Re-reconstruct subtomograms at 5 Å /px
3D Refinement, Relion
Subtomos. at 2.5 Å /px
3D Classification, Relion
Class I
29,656 pctls
Class II
408 pctls
Class III
1,732 pctls
Class IV
337 pctls
Subtomos. at 1.6 Å /px
3D Refinement, Relion
3D Classification
Relion
Class I
2,801 pctls
Class II
3,832 pctls
Class III
738 pctls
Class IV
22, 254 pctls
3D Refinement in M, followed by Relion
B
180º
C
Gas
Water
Water
Gas
D
Global resolution = 7.3 Å
Fourier Shell Correlation
1.0
0.143
1.0
0.00
0.05
0.10
0.15
0.20
0.25
Frequency (1/Å)
X
Y
Z
Global

Figure S5. CryoET data processing for AnaS GV shell, related to Figure 3 and STAR Methods. (**A**) Simplified schematic of the subtomogram averaging workflow highlighting crucial steps in the pipeline. (**B**) Orthogonal views of the final average. (**C**) Gray-scale orthographic slices of AnaS GV shell in the positions indicated by the blue and orange dashed lines in **B**. (**D**) Global FSC curve for AnaS GV shell (yellow), and map anisotropy analysis by FSC curves in X (pink), Y (purple) and Z (blue) directions.

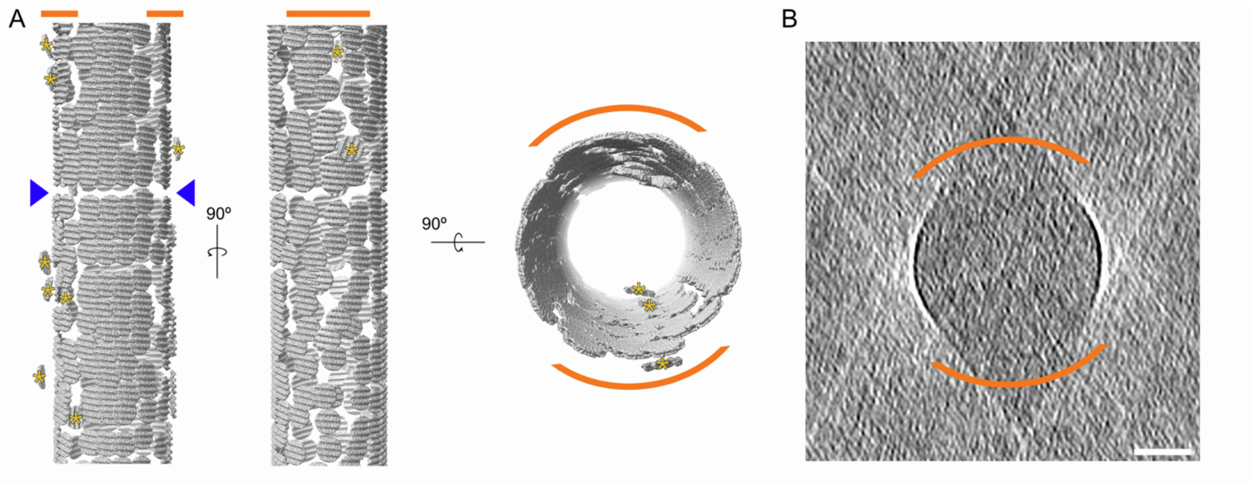

Figure S6. Particle poses, related to Figure 3 and STAR Methods. (**A**) Orthogonal views of the particle orientations after subtomogram averaging for a representative tomogram of native Ana GV. Blue arrows indicate the position of the inversion point. Misaligned subtomograms are marked with yellow asterisks. (**B**) Tomographic slice showing that the missing wedge orientation corresponds with the most distorted particles. Scale bar, 25 nm. Orange lines indicate position of the missing wedge.

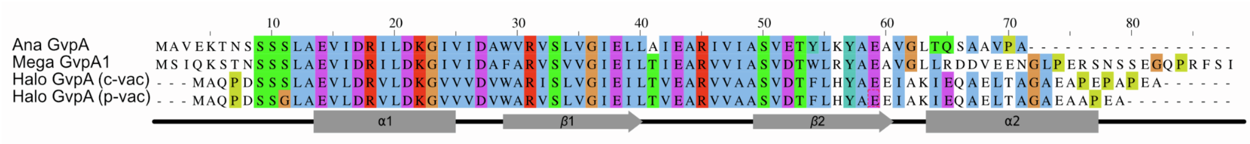

Figure S7. Protein sequence alignment, related to Figure 4. Sequence alignment among homologs of the major structural protein (GvpA) from Mega, Ana, and Halo (p-vac and c-vac gene clusters).

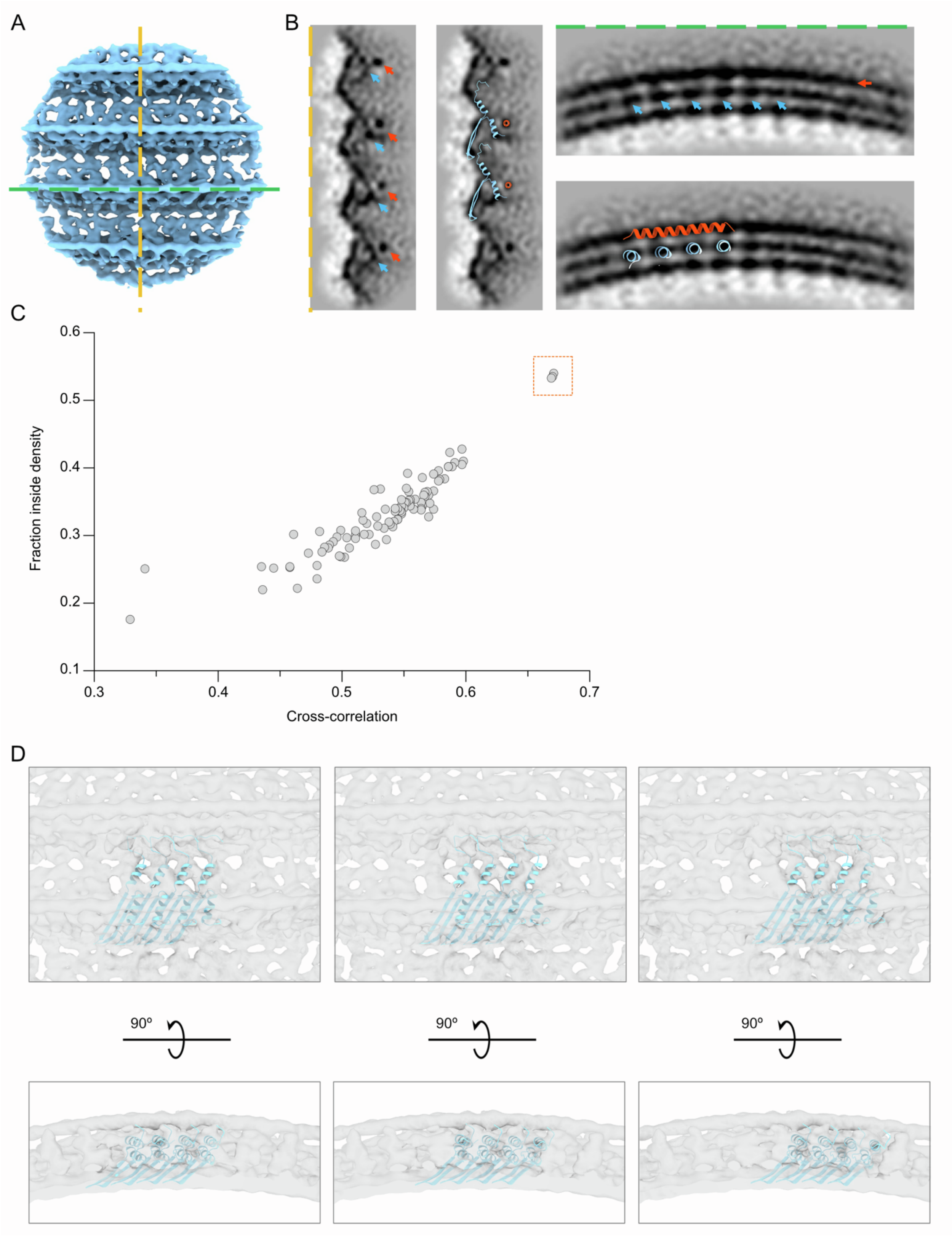
A
B
C
D
Fraction inside density
Cross-correlation
0.1
0.2
0.3
0.4
0.5
0.6
0.3
0.4
0.5
0.6
0.7
90º
90º
90º

Figure S8. Model fitting validation, related to Figure 4, 5 and STAR Methods. (**A-B**) Visual inspection of secondary structures matching with cryoET density. (**A**) Isosurface rendering. Dashed lines indicate positions of the orthographic slices visualized in **B**. (**B**) Gray-scale orthographic slices of the native Ana GV shell. Secondary structures of GvpA and GvpC are overlayed with the cryoET density visible in orthographic slices. Orange arrows indicate position of the GvpC and blue arrows positions of the GvpA fragments that bind GvpC. (**C**) Results of the rigid-body fitting of four GvpA molecules in Chimera using “fitmap” command. Three best scoring results are highlighted by the orange dashed box. (**B**) The three best results with similar fitting scores (orange dashed box in panel A) all fit our density map. Each fit from left to right are only different in that they shift by one subunit along Y-axis.

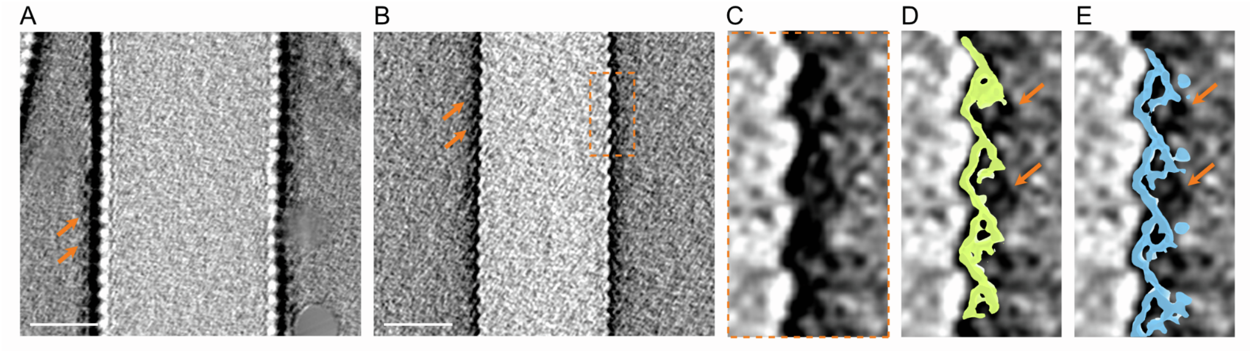

Figure S9. Additional densities on the surface of Mega GVs, related to Figure 4. (**A**,**B**) Slices from cryo-electron tomograms of individual Mega GVs show additional density on the surface. Defocus values: (**A**) -5 µm and (**B**) -1 µm. (**C**) Enlarged section form **B** as outlined by orange dashed box. (**D, E**) Superimposition of subtomogram averages (Figure 3C and D) for AnaS and Ana GV shell. Orange arrows indicate extra densities. Scale bars, 20 nm.

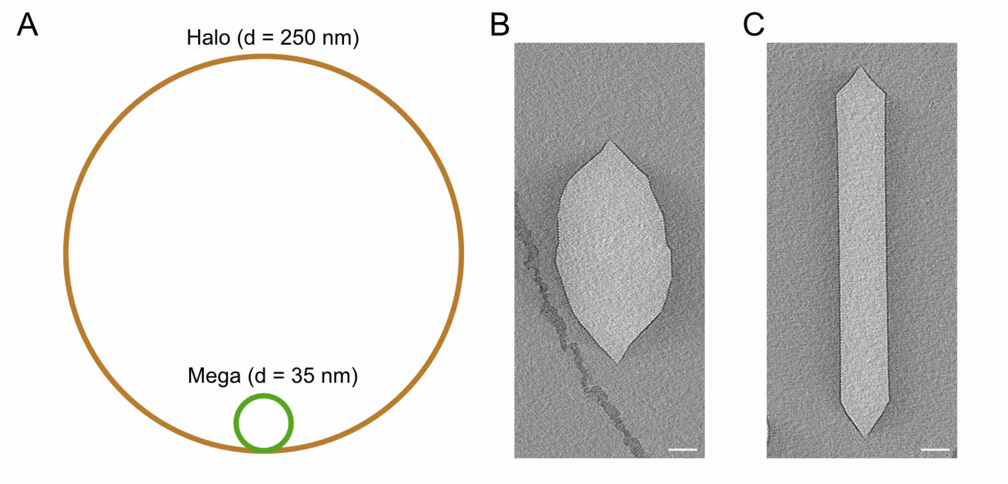

Figure S10. GVs adopt a wide range of diameters and different morphologies, related to Figure 4. (**A**) Schematic showing difference in rib curvature between smallest (Mega) and largest (Halo) measured diameter (Dutka et al. 2021). (**B**,**C**) Representative central slices from cryo-electron tomograms of individual Halo GVs encoded by (**B**) p-vac and (**C**) c-vac gene clusters. Scale bars, 50 nm.

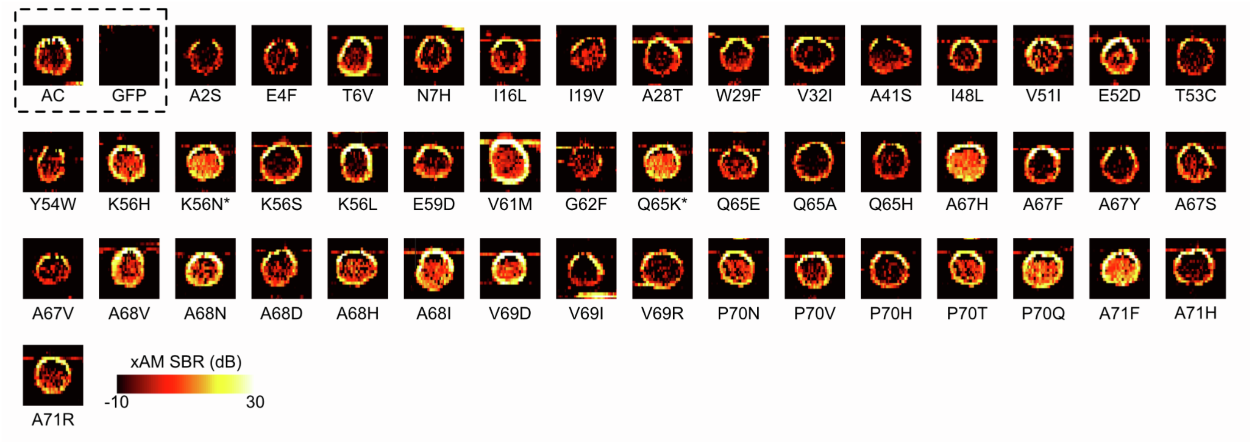

Figure S11. Ultrasound images of *E. coli* clones expressing select GvpA mutants, related to Figure 4. Pre-minus-post-collapse nonlinear xAM images of clones of *E. coli* expressing GVs with the indicated mutations in GvpA. All the shown mutants display clear non-zero contrast and therefore successfully form GVs. Wild type GvpA (AC) and GFP are included as positive and negative controls, respectively. Color map corresponds to SBR, the signal-to-background ratio. *Mutations K56N and Q65K occurred in the same clone. GV expression is more pronounced on the edges of the patches because of those cells' increased access to nutrients.

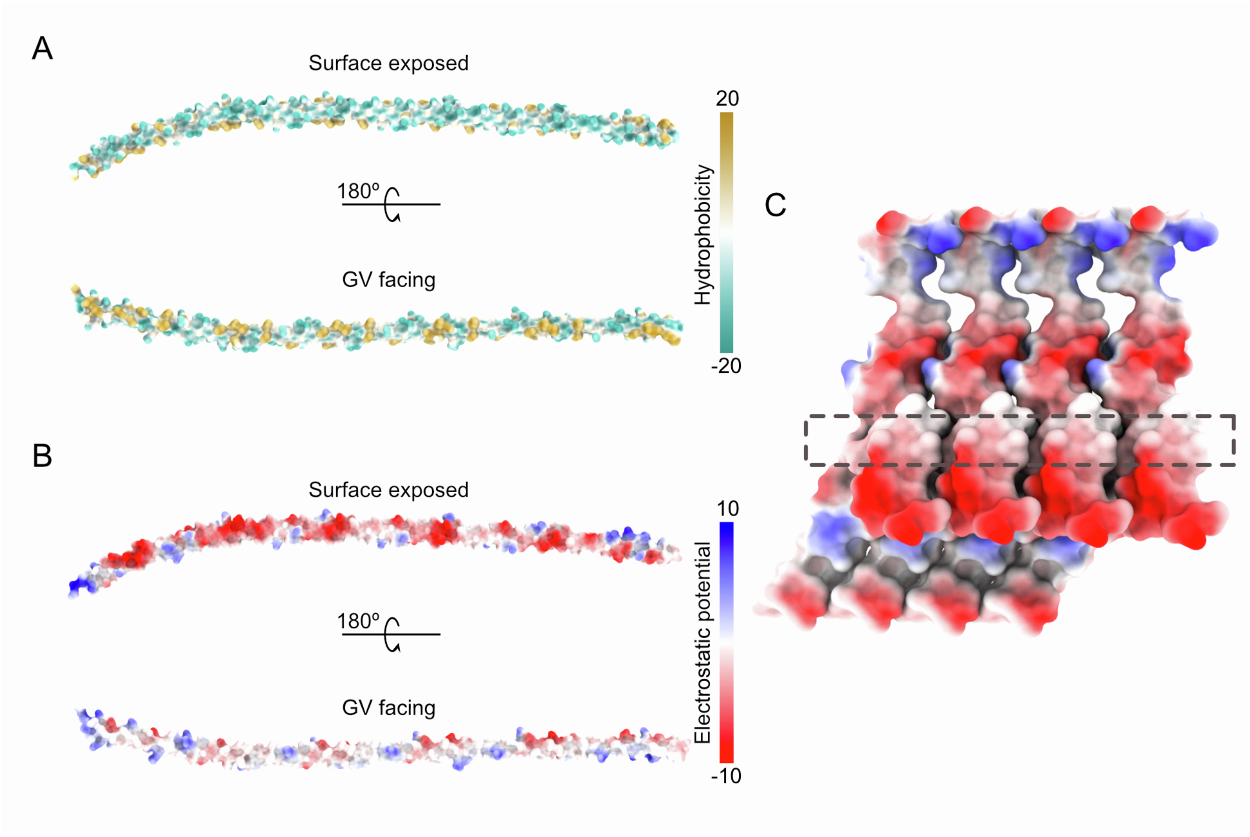

Figure S12. Hydrophobicity and charge distribution on GvpC surface, related to Figure 5. (**A**,**B**) AlphaFold2 predicted models of full-length GvpC**.** (**A**) Hydrophobicity of the GvpC surface. (**B**) Distribution of the electrostatic potential of the GvpC surface. (**C**) Distribution of the electrostatic potential on the GvpC binding model.

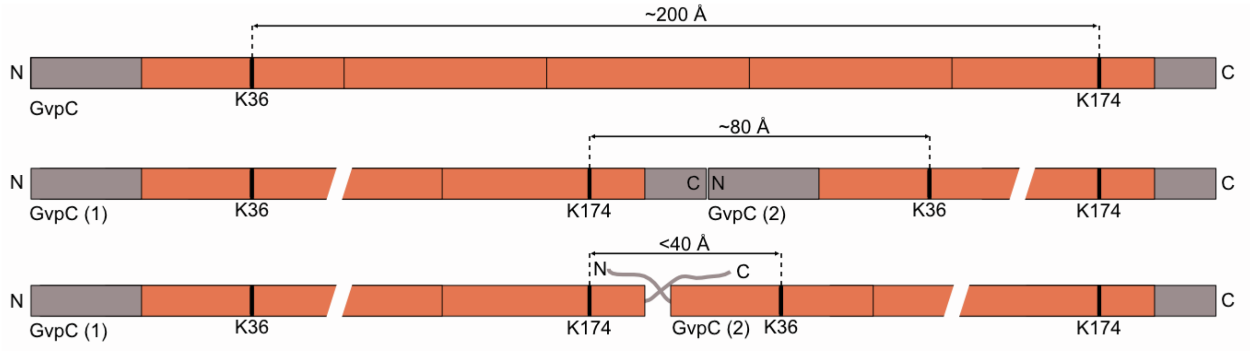

Figure S13. Distances for different scenarios of Lys cross-linking between GvpC molecules, related to Figure 5.

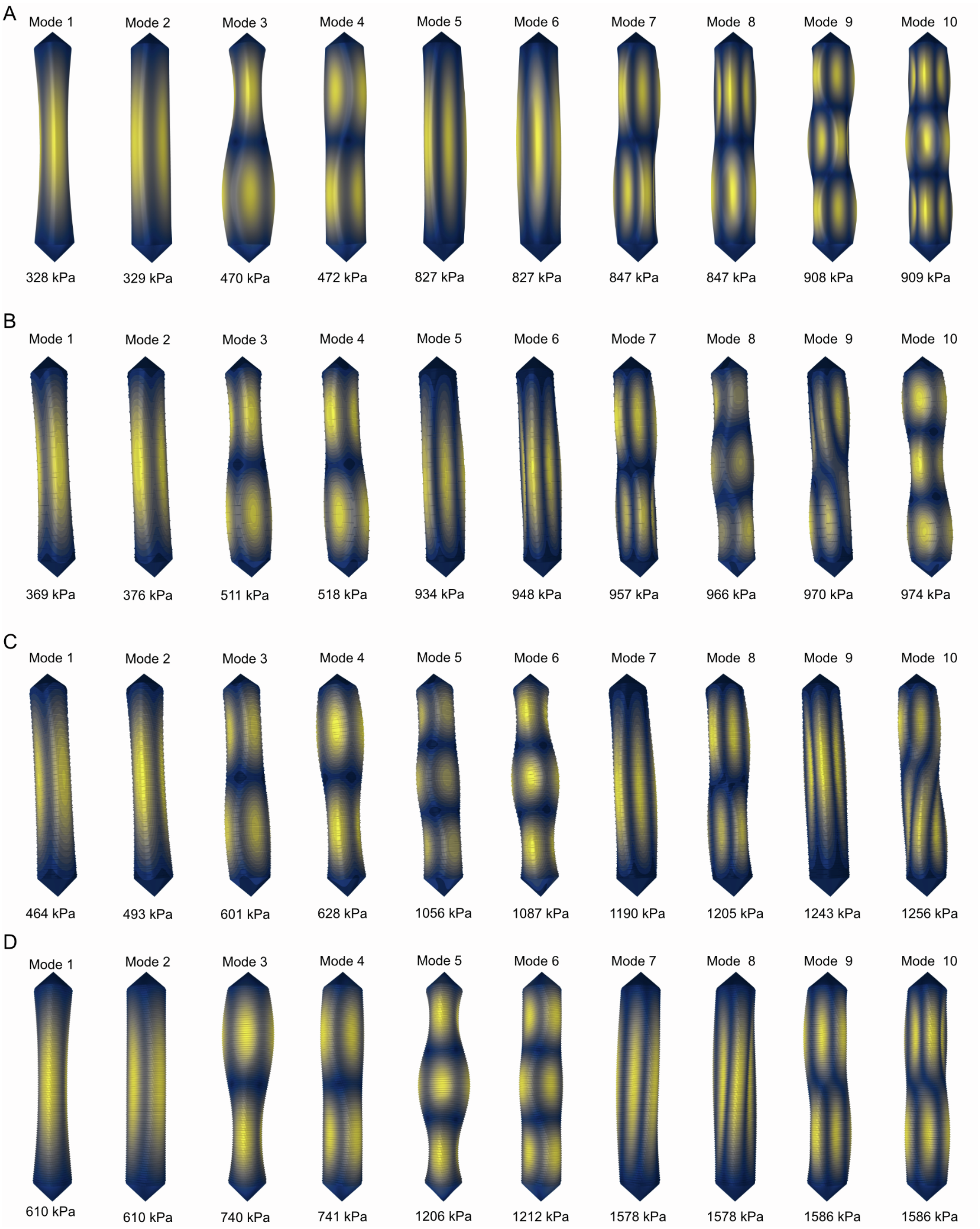

Figure S14. Buckling modes of GVs with different degrees of GvpC saturation, related to STAR Methods. The first ten buckling modes and pressures were obtained from linear buckling analysis for GV with distinct saturation levels of GvpC. Rows from top to bottom represent GvpC densities of (**A**) 0%, (**B**) 20%, (**C**) 60%, and (**D**) 100%.

Table S1. Data collection and processing parameters for GV shell, related to Figure 3 and STAR Methods.

	Native GVs	AnaS GVs
Magnification	53,000×	53,000×
Voltage (keV)	300	300
Energy Filter	Yes	Yes
Slit width (eV)	20	20
Pixel size (Å)	0.8435	0.8435
Defocus range (μm)	1.5 to 3.5	1.5 to 3.5
Defocus step (μm)	0.5	0.2
Tilt range (min/max, step)	-60/60°,3° or -44/44°,4°	-45/45°,3°
Tilt scheme	Dose-symmetric	Dose-symmetric
Total dose (electrons/Å^2)	~45	~45
Frame number	10 or 5	10
Tomograms used/acquired	127/368	28/103
Number of cylinders	136	32
Final subtomograms (no.)	73,866	28,887
Symmetry	C1	C1
Map resolutions (FSC = 0.143)	7.7 Å	7.3 Å

Table S2. List of validated cross-linked peptides, related to Figure 5 and STAR Methods.

**Cross-linker type**	Protein 1	Sequence 1	Protein 2	Sequence 2	Cross-linked residues	Score
Cross-links						
BS^3	GvpC	**M**ISLMAK	GvpA	**M**AVEK	0-0	31.72
BS^3	GvpA	MAVE**K**TNSSSSLAEVIDR	GvpC	ISLMA**K**IR	5-7	53.19
DSSO	GvpC	QEHQSIAE**K**VAELSLETR	GvpA	**A**VEK	18-1	65.91
DSSO	GvpC	EFLSVTTA**K**R	GvpA	**A**VEK	36-1	60.36
DSSO	GvpC	IAQAE**K**QAQELLAFYQEVR	GvpA	**A**VEK	109-1	58.99
DSSO	GvpC	TAQA**K**EQK	GvpA	**A**VEK	174-1	43.9
Cross-links (inter- or intra- molecular)						
DSSO	GvpC	QEHQSIAE**K**VAELSLETR	GvpC	EFLSVTTA**K**R	18-36	102.98
DSSO	GvpC	TAQA**K**EQK	GvpC	ESLL**K**FR	174-182	86.63
DSSO	GvpC	EFLSVTTA**K**R	GvpC	TAQA**K**EQK	36-174	84.36
DSSO	GvpC	QEQAE**K**QAQELQAFYK	GvpC	EFLSVTTA**K**R	43-36	71.03
DSSO	GvpA	**M**AVEK	GvpA	**A**VEK	0-1	49.79
DSSO	GvpA	ILD**K**GIVIDAWVR	GvpA	**A**VEK	22-1	58.99
